# Supplementary material for: Guided self-help treatment for children and young people with threshold and subthreshold eating disorders: A pilot study protocol
Source: PLoS One. 2024 Apr 16;19(4):e0301606. doi: 10.1371/journal.pone.0301606 (PMC11020482; doi:10.1371/journal.pone.0301606)
Supplement: S1 Appendix — (DOCX) [file pone.0301606.s001.docx]

# Appendix 1: Model consent form

**Project title: Short Psychological Intervention for Children and adolescents with Eating disorders (SPICE)**

**CHILD AND YOUNG PERSON (AGED 16-19) CONSENT FORM**

**1 copy for participant; 1 copy for researcher**

**Please complete this form after you have read the Information Sheet and/or listened to an explanation about the research.**

Thank you for considering taking part in this research. The person organising the research must explain the project to you before you agree to take part. If you have any questions arising from the Information Sheet or explanation already given to you, please ask the researcher before you decide whether to join in. You will be given a copy of this Consent Form to keep and refer to at any time.

**I confirm that I understand that by initialling each box below I am consenting to this element of the study. I understand that it will be assumed that un-initialled boxes means that I DO NOT consent to that part of the study. I understand that by not giving consent for any one element that I may be deemed ineligible for the study.**

|  |  | *Please initial all boxes* |
| --- | --- | --- |
| 1. | I confirm that I have read and understood the information sheet (21/09/2023; version 3) for the above study. I have had the opportunity to consider the information, ask questions and where appropriate, have had these answered satisfactorily. |  |
| 2. | I understand that my participation is voluntary and that I am free to withdraw at any time without giving any reason, without my medical care or legal rights being affected. |  |
| 3. | In the event that I decide to stop taking part in the study, I agree to the information that I have provided up to that point to be used in the analysis of the study results. |  |
| 4. | I agree to allow information I provide about myself to be entered on a confidential computer database held at the Institute of Child Health, University College London. |  |
| 5. | I consent to the interviews and guidance sessions being audio/video-recorded and understand that the recordings will be stored anonymously, using password-protected software. I give permission for this to happen. |  |
| 6. | I give permission for my direct quotes to be used anonymously in any resulting reports, publications and presentations. |  |
| 7. | I agree to my General Practitioner (GP) being informed of my participation in the study. |  |
| 8. | I agree to my GP being sent copies of the written reports that will summarise the outcomes from the assessments conducted at the start of the study and at the end of the intervention. |  |
| 9. | If you are currently under the care of an eating disorder service (e.g., you are on the waitlist for an assessment or treatment): I agree to the eating disorder service being sent copies of the written reports that will summarise the outcomes from the assessments conducted at the start of the study and at the end of the intervention. |  |
| 10. | In the event that I disclose any information that suggests a risk of harm to myself or others, I agree for this information to be disclosed to the relevant bodies, including my GP. |  |
| 11. | I understand the potential risks of participating and the support that will be available to me should I become distressed during the course of the research. |  |
| 12. | I agree to take part in the above study. |  |
| 13. | ***Optional. I give permission for my contact details to be retained so that the research team can contact me about future research. |  |

Name of participant Signature Date

______________________________ ________________________ _______________

Name of person taking consent Signature Date

______________________________ ________________________ _______________
