# Supplementary material for: Guided self-help treatment for children and young people with threshold and subthreshold eating disorders: A pilot study protocol
Source: PLoS One. 2024 Apr 16;19(4):e0301606. doi: 10.1371/journal.pone.0301606 (PMC11020482; doi:10.1371/journal.pone.0301606)
Supplement: S1 File — (DOCX) [file pone.0301606.s004.docx]

**Study title: Short Psychological Intervention for Children and adolescents with Eating disorders (SPICE)**

**Protocol number:** 23PP09

**Protocol version:** 4

**IRAS ID:** 323971

**Chief investigator:** Professor Roz Shafran

**Funder:** UCL Great Ormond Street Institute of Child Health

**Sponsor:** UCL Great Ormond Street Institute of Child Health, 30 Guilford Street, London, WC1N 1EH, United Kingdom

**Signatures**

The Chief Investigator, Principal Investigators and Sponsor have discussed this protocol. All have agreed to perform the investigation as written and to abide by this protocol except in case of medical emergency or where departures from it are mutually agreed in writing.

**Chief investigator**

Signature:

Date: 17/10/2023

**Amendment history**

| **Amendment no.** | **Protocol version no.** | **Date issued** | **Author(s) of changes** | **Details of changes made** |
| --- | --- | --- | --- | --- |
| 1 | 3 | 11/10/2023 | Emily Davey | This amendment seeks to make the following changes:  (i) to collect CYP date of birth and address at baseline; to collect CYP weight throughout treatment  (ii) to deliver the intervention via an online website and interactive PDFs  (iii) to change outcome measure from ChEDE (interview) to EDE-Q (questionnaire)  (iv) to change the optional item of sharing the outcomes of the intervention with an eating disorder service on the consent form to non-optional  (v) to access existing data from services to have same data for everyone  (vi) to make changes to the information sheets in line with changes listed above and other minor changes  (vii) to make changes to the protocol in line with changes listed above and other minor changes |
| 2 | 4 | 17/10/2023 | Emily Davey | This amendment seeks to make the following changes:  (i) to add a parent-reported measure of child eating difficulties (PEDE-Q) at baseline and post-intervention |

**Abbreviations**

| CBT | Cognitive Behaviour Therapy |
| --- | --- |
| CI | Chief Investigator |
| CYP | Children and Young People |
| DSH | Data Safe Haven |
| FBT | Family-Based Therapy |
| GP | General Practitioner |
| GSH | Guided Self-Help |
| ICH | UCL Great Ormond Street Institute of Child Health |
| RCT | Randomised Controlled Trial |

**Study summary**

| Title | Short Psychological Intervention for Children and adolescents with Eating disorders (SPICE) study |
| --- | --- |
| Sponsor name | UCL Great Ormond Street Institute of Child Health |
| Primary objective | To evaluate the feasibility, acceptability and preliminary effectiveness of a guided self-help (GSH) intervention for children and young people (CYP) with eating disorders |
| Study design | Case series |
| Study endpoints | The final follow-up measurement (4 weeks post-intervention) for the final participant |
| Sample size (maximum) | Aim to recruit 10 CYP and their parents/carers |
| Summary of eligibility criteria | CYP  *Inclusion criteria:*   - Aged 8-19 years - Has a threshold or sub-threshold eating disorder (anorexia nervosa, bulimia nervosa, binge eating disorder, other specified feeding and eating disorder [OSFED]) which impairs their psychosocial functioning - UK resident - Has a parent/carer willing to take part in the study   *Exclusion criteria:*   - Does not speak/understand English sufficiently to access the measures and intervention materials - Has an intellectual disability at a level meaning that they cannot access the measures and intervention materials - Acute risk not considered suitable for the study due to the clinical need for immediate and/or specialist intervention (e.g., rapid weight loss, very low mood, high medical or psychiatric risk, acute suicidality, recurrent or potentially life limiting self-harm and/or significant safeguarding concerns) - Unstable psychotropic medication dosage - Receiving other overlapping psychological support - Does not have access to a telephone/computer which can be used for the assessment and guidance sessions   Parents/carers  *Inclusion criteria:*   - Parent/legal guardian of the CYP - UK resident   *Exclusion criteria:*   - Does not speak/understand English sufficiently to access the measures and intervention materials - Has an intellectual disability at a level meaning that they cannot access the measures and intervention materials |
| Intervention | CBT-based Self-help with 8 guidance sessions over 8 weeks |
| Procedures: Screening & enrolment | Families at specialist eating disorder services will be approached by a member of their clinical care team, and eligibility will be determined by a screening questionnaire and an eating disorder questionnaire (Eating Disorder Examination Questionnaire [EDE-Q]) |
| Baseline and outcome measures | Baseline and post-intervention measures:   - Eating Disorder Examination Questionnaire self-report (EDE-Q) and parent-report (PEDE-Q) - Clinical Impairment Assessment (CIA) - %Weight-for-height - Revised Child Anxiety and Depression Scale (RCADS) - DAWBA (baseline only) or SDQ (total and impact supplement)   Session-by-session measures:   - Eating Disorder-15 for Youth (ED-15-Y) - Goal Based Outcomes (GBOs) - Outcome Rating Scale (ORS)/Child Outcome Rating Scale (CORS) - SDQ Session-by-session (SDQ SxS)   Post-intervention measures only:   - Acceptability questionnaire - Optional qualitative interview |
| Treatment period | 3 months maximum |
| End of study | The final post-intervention assessment (12 weeks after baseline measures) for the final participant or the last qualitative interview with the final participant, whichever occurs last |

**Key study contacts**

| Chief Investigator | Professor Roz Shafran  UCL Great Ormond Street Institute of Child Health, 30 Guilford Street, London, WC1N 1EH  [r.shafran@ucl.ac.uk](mailto:r.shafran@ucl.ac.uk)  020 7242 9789 |
| --- | --- |
| Study Co-ordinator | Emily Davey  UCL Great Ormond Street Institute of Child Health, 30 Guilford Street, London, WC1N 1EH  [emily.davey.21@ucl.ac.uk](mailto:r.shafran@ucl.ac.uk)  07500 115 165 |
| Sponsor | UCL Great Ormond Street Institute of Child Health, 30 Guilford Street, London, WC1N 1EH  020 7242 9789 |
| Funder | UCL Great Ormond Street Institute of Child Health, 30 Guilford Street, London, WC1N 1EH  020 7242 9789 |

TABLE OF CONTENTS

[Rationale and background information 7](#_Toc129343485)

[Objective and purpose 8](#_Toc129343486)

[Theoretical framework 8](#_Toc129343487)

[Methods 9](#_Toc129343488)

[Study setting 9](#_Toc129343489)

[Study design 9](#_Toc129343490)

[Participants 10](#_Toc129343491)

[Procedure 11](#_Toc129343492)

[Measures 15](#_Toc129343493)

[Data analysis 20](#_Toc129343494)

[Intervention 21](#_Toc129343495)

[Discontinuation/Withdrawal of participants from study 22](#_Toc129343496)

[Ethical and safety considerations 23](#_Toc129343497)

[Risk management 26](#_Toc129343498)

[Data recording and record keeping 26](#_Toc129343499)

[Confidentiality and information governance 26](#_Toc129343500)

[Dissemination of results and publication policy 28](#_Toc129343501)

[Financial information and insurance 28](#_Toc129343502)

[References 29](#_Toc129343503)

[Appendices 34](#_Toc129343504)

*The current protocol relates to a study that is part of a wider PhD project which sets out to design and evaluate a low-intensity, transdiagnostic psychological treatment for children and young people with eating disorders.*

# Rationale and background information

Eating disorders are disabling conditions that markedly impair physical health and disturb psychosocial functioning (Treasure, 2020). They have high psychiatric and medical comorbidity, and one of the highest rates of mortality among mental health disorders (van Hoeken & Hoek, 2020). Eating disorders can impact substantially on an individual’s health-related quality of life, and are associated with elevated healthcare utilisation and significant economic costs (Ágh et al., 2016; Santomauro et al., 2021). They often begin during adolescence, and are prevalent among those who present to child and adolescent mental health services in the UK (National Collaborating Centre for Mental Health, 2015).

Despite the seriousness of these disorders, access to care for children and young people (CYP) with eating disorders has long been challenging, and this has been exacerbated further by the COVID-19 pandemic (Spigel et al., 2021). The COVID-19 pandemic has adversely affected CYP with eating disorders, with an increased incidence of a first diagnosis and deteriorating symptoms among those with pre-existing diagnoses (Katzman, 2021). Child and adolescent eating disorder services in the UK have seen almost a doubling in the number of referrals (NHS England, 2023), and it is estimated that 60% of young people aged 17-19 have disordered eating (Newlove-Delgado, 2022). NHS services have struggled to meet this increased demand for treatment, with only 68% of urgent and 69% of routine cases seen within the standard (a target of 95%) during April-June 2022 (National Audit Office, 2023). This is concerning given that delays in receiving specialist treatment can increase the risk of chronicity and burden of illness (Striegel Weissman & Rosselli, 2017). This demand-capacity gap highlights the urgent need for less resource-intensive interventions which are scientifically supported, accessible and scalable for this patient group, such as guided self-help.

Guided self-help interventions are the first step treatment for adults with bulimia nervosa and binge eating disorder (National Institute for Health and Care Excellence, 2017), and have proven efficacy for CYP with anxiety and depression (Bennett et al., 2019; Thirlwall et al., 2013), which often co-occur with eating disorders (Keski-Rahkonen & Mustelin, 2016). However, child and adolescent eating disorder services do not routinely use guided self-help interventions as they have not been sufficiently researched. The limited research that does exist suggests that guided self-help interventions have the potential to be effective for this patient group. In their randomised controlled trial (RCT), Schmidt et al. (2007) compared cognitive behavioural therapy (CBT) guided self-care with family therapy in 85 adolescents with bulimia nervosa and related disorders, and the outcomes indicated no difference between groups, with the guided self-care showing greater impact at 6 months and being more cost-effective. Lock et al. (2021) conducted a pilot RCT of 40 adolescents with anorexia nervosa which compared online family-based guided self-help to family-based treatment delivered via videoconferencing. The results suggested that family-based guided self-help was acceptable to families and led to improvements in terms of both weight gain and global eating disorder psychopathology. However, both of these interventions remain in the domain of research and are not readily available for CYP with eating disorders in the UK.

# Objective and purpose

The overarching aim of this project is to improve access to psychological treatments for CYP affected by eating disorders. Specifically, this preliminary study aims to examine the feasibility, acceptability and preliminary effectiveness of a guided self-help intervention for CYP (aged 8-19 years) with impairing symptoms of eating disorders.

# Theoretical framework

The development and evaluation of this intervention will be considered within the Medical Research Council’s (MRC) updated framework for the development and evaluation of complex interventions (Skivington et al., 2021). The framework divides research of complex intervention into four distinct, but not necessarily sequential, phases: development/identification of the intervention; feasibility; evaluation; and effective implementation (see Figure 1 below). This study will predominantly focus on the feasibility and preliminary evaluation aspects of this framework.

**Feasibility**

Assessing feasibility and acceptability of intervention and evaluation design in order to make decisions about progress to next stage of evaluation

**Evaluation**

Assessing an intervention using the most appropriate method to address research questions

**Implementation**

Deliberate efforts to increase impact and uptake of successfully tested health interventions

**Core elements**

- Consider context
- Develop, refine and (re)test programme theory
- Engage stakeholders
- Identify key uncertainties
- Refine intervention
- Economic considerations

**Develop intervention**

Developing a new intervention, or adapting an existing intervention for a new context based on evidence base and theory of the problem

**Identify intervention**

Choosing an intervention that already exists (or is planned), either via policy or practice, and exploring its options for evaluation (evaluability assessment)

**OR**

Figure 1: Medical Research Council Framework for the development and evaluation of complex interventions (Skivington et al., 2021)

# Methods

## Study setting

The main research sites identified for this project are specialist child and adolescent eating disorder services across the UK.

## Study design

This study will employ a case-series design in which CYP with impairing eating disorder symptoms, and their parents/carers, will receive a self-help intervention and 8 x 30-minute guidance sessions via videocall or telephone over a period of 8 weeks. Standardised assessments will be administered at baseline and posttreatment, and on a weekly basis throughout the intervention. The feasibility, acceptability and preliminary treatment effects will be specifically examined.

## Participants

This study will look to recruit CYP (aged 8-19 years) with impairing symptoms of eating disorders, and their parents/carers. In line with previous case series research (da Luz et al., 2017; Dumont et al., 2019), the aim will be to recruit approximately 10 eligible families to receive the CBT guided self-help intervention.

**Eligibility criteria**

### Children and young people

Inclusion criteria

The inclusion criteria for CYP:

1. Aged 8-19 years
2. Has a threshold or sub-threshold eating disorder (anorexia nervosa, bulimia nervosa, binge eating disorder and otherwise specified feeding and eating disorder [OSFED]) which impairs their psychosocial functioning
3. Is a UK resident
4. Have a parent/carer who is also willing to take part in the study

Exclusion criteria

The exclusion criteria for CYP:

1. Does not speak/understand English sufficiently well to access the measures and intervention materials. This is primarily due to limited funding capacities for interpreters
2. Has an intellectual disability at a level meaning that they cannot access the measures and/or intervention. This will not be defined by IQ, but by clinical judgement. CYP will not be excluded because of the presence of intellectual disability per se, but because of being unable to access the materials. Ability to participate may be different for younger children who are less able, whose parents complete the materials, compared to older children who need to complete the materials themselves. If the intellectual disability is identified during the initial assessment (through clinical judgement), young people will be referred to other more appropriate services as necessary (with agreement of the family)
3. Questionnaire indicates acute risk not considered suitable for the trial due to the clinical need for immediate and/or specialist intervention, e.g., rapid weight loss, very low mood, high medical or psychiatric risk, acute suicidality, recurrent or potentially life limiting self-harm and/or significant safeguarding concerns (i.e., if the child has a child protection plan and/or is on the child protection register, and/or the research team consider the child to be suffering, or likely to suffer, significant harm)
4. If they have been prescribed psychotropic medication, the dosage must have been stable for the past two months
5. Currently receiving other overlapping psychological support/interventions
6. Does not have access to a telephone or laptop/computer which can be used for the interviews and guidance sessions

### Parent/carers

Inclusion criteria

The inclusion criteria for parents/carers:

1. Is a parent/legal guardian of the CYP
2. Is a UK resident

Exclusion criteria

The exclusion criteria for parents/carers:

1. Does not speak/understand English sufficiently well to access the measures and intervention materials. This is primarily due to limited funding capacities for interpreters
2. Has an intellectual disability at a level meaning that they cannot access the measures and/or intervention. This will not be defined by IQ, but by clinical judgement. Parents/carers will not be excluded because of the presence of intellectual disability per se, but because of being unable to access the materials

## Procedure

### Recruitment and informed consent of participants

Specialist child and adolescent eating disorder services in the UK have been identified as research sites for this project. Emily Davey will obtain honorary contracts at each research site for research activity.

CYP, and their parents/carers, will be contacted by a member of their clinical care team at the site in question. The member of the patient’s clinical care team will provide an overview of the study and will ask families if they would like to receive the study information (i.e., participant information sheet).

If the family agree to receive a participant information sheet, the member of the clinical team will provide this to the family (either electronically or in a paper copy). The information sheet will contain the contact details of the research team so that the CYP, and/or their parents/carers, can contact the research team to ask any questions they may have. The information sheet will also contain a QR code to an online form that potential participants can fill in to share their contact details with the research team to enable the research team to contact them directly.

The member of the clinical team will also ask families for explicit written or verbal consent for their contact details to be held by the research team going forwards. The member of the clinical team will sign a ‘consent to be contacted by the research team’ form to confirm whether written or verbal consent has been obtained by the family for their contact details to be held by the research team.

After a reasonable period of time, the family will be contacted by the study researcher and asked if they have any questions about the study and/or are interested in taking part. Once they have decided to take part, the researcher will gain informed consent/assent via an online consent/assent form. Parents/carers and CYP (aged ≥ 16) will be asked to provide informed consent to participate in the study, and CYP (aged < 16) will be asked to provide assent, in addition to the consent of their parent/carer.

All CYP, and their parents/carers, who start the treatment will be asked by the study researcher if they would like to take part in an optional qualitative interview to explore their experiences and views of the treatment approach. CYP, and their parents/carers, will be able to ask the study researcher any questions they may have. If they are interested in taking part, they will be asked to complete an online consent/assent form.

### Screening and eligibility assessment

Once families have been recruited through one of the recruitment sites, and have completed the study consent/assent forms, their eligibility for the research will be confirmed.

Families who consent/assent to participate in the study will be asked to complete a short screening questionnaire to determine their eligibility for participation (see [Appendix 1](#Appendix1)). This will include demographic information about the CYP and their parent(s)/carer(s), confirmation of UK residency, information about prescribed psychotropic medication of the CYP, current/previous psychological support received by the CYP, brief questions to determine if the CYP is experiencing possible symptoms of eating disorders that impair their functioning and a question around current self-harm and suicidality. This questionnaire can be completed online or via telephone with the study researcher. It is expected that this questionnaire will take approximately 15 minutes to complete. If families are not eligible for the study, the study researcher will contact the family to explain why they are not eligible for the research, and signpost them to relevant support, as necessary. If the screening questionnaire indicates that the family meet the eligibility criteria, they will be invited to enrol in the study.

### Procedure once participants are enrolled

Once participants are enrolled into the study, CYP and their parents/carers will be invited to complete the baseline measures, including a battery of questionnaires, including the self-report of the Eating Disorder Examination Questionnaire [EDE-Q] and the parent-report of Eating Disorder Examination Questionnaire [PEDE-Q]). This is to confirm that the intervention is appropriate (i.e., that the CYP has impairing symptoms of eating disorders and an absence of significant risk as detailed in the inclusion/exclusion criteria). Families may also be asked to complete the online Development and Wellbeing Assessment (DAWBA) to enable an understanding of comorbid diagnoses. Note – the DAWBA will be piloted with families in the first instance. If the family have already completed the DAWBA during their assessment with the eating disorder service, this data will be accessed to prevent duplication of measures. Following the pilot, it may be decided to no longer use the DAWBA, and families will be asked to complete the Strengths and Difficulties Questionnaire (SDQ) at baseline instead.

Questionnaires will be sent to families to complete online (or over the telephone if preferred). The questionnaires will cover some additional demographic information about the family ([Appendix 2](#Appendix2)), brief questions about the CYP’s eating disorder symptoms and how they impair their functioning, and symptoms of anxiety and depression. They will also be asked to report on the CYP’s current weight and height. It is expected that these questionnaires will take approximately 15 minutes to complete. Families may also complete the DAWBA online (or over the telephone with a researcher if preferred) which will take around 45 minutes to complete.

Families will then be invited to attend a feedback appointment where the study researcher will feed back the information obtained from the questionnaires, and to discuss next steps regarding the intervention. This feedback appointment is likely to last around 30 minutes. The family will be given a report summarising the outcomes of the questionnaires and the next steps for treatment. The family’s GP and other teams involved in their care (e.g., eating disorder service) will also be sent a copy of this report to inform them about the family’s participation in the study.

Families will commence treatment up to one week after completing the baseline measures (depending on how soon they can attend their first guidance session). Families will be given access to a website and interactive PDFs, and will receive 8 individual guidance sessions via a videocall or over the telephone with a Guide. They will be asked to complete weekly questionnaires throughout the intervention to assess week-to-week changes. These weekly questionnaires will take around 15 minutes to complete each week.

CYP and their parents/carers will be asked to complete the post-treatment measures 12 weeks after completing the baseline measures. This involves reporting on the CYP’s current weight for height, completing a brief questionnaire about the CYP’s symptoms of eating disorder, anxiety and depression, and emotional and behavioural difficulties. It will also involve a questionnaire to assess the acceptability of the treatment approach. It is anticipated that these questionnaires will take approximately 30 minutes to complete.

Families will then be invited to attend a feedback appointment to review the outcomes of these post-treatment measures, and to signpost them to further support if required. This appointment is expected to be around 30 minutes in duration.

CYP, and their parents/carers, will also be invited to attend an optional qualitative interview to explore their experiences of the intervention. It is anticipated that this interview will last approximately 45 minutes.

## Measures

Screening measures

Families will be asked to complete initial screening questions to determine their eligibility for the research and to describe the study sample. This will include demographic information about the CYP and their parent(s)/carer(s), confirmation of UK residency, information about prescribed psychotropic medication of the CYP and current/previous psychological support received by the CYP. They will also be asked to complete a brief questionnaire to determine if the CYP is experiencing possible symptoms of eating disorders (i.e., the Child Eating Disorder Examination Questionnaire – 8-items; ChEDE-Q8), as well as some questions regarding the extent to which these eating disorder symptoms impair functioning (adapted from the Strengths and Difficulties Questionnaire (SDQ) impact supplement) and a question around current risk (see [Appendix 1](#Appendix1)).

*Child Eating Disorder Examination Questionnaire – 8-items (ChEDE-Q8; Kliem et al., 2017)*

The ChEDE-Q8 is an 8-item self-report measure that evaluates four areas of eating disorder psychopathology: restraint, eating concern, shape concern and weight concern. The ChEDE-Q8 scores range from 0 (absence of the feature) to 6 (feature present every day to an extreme degree). Global mean scores will be calculated, with higher scores indicating higher levels of eating disorder psychopathology. The ChEDE-Q8 has been validated for children aged ≥ 7 years and has been shown to have strict measurement invariance, internal consistence as well as convergent and factorial validity (Kliem et al., 2017).

Baseline only measures

Psychiatric diagnoses

*The Development and Wellbeing Assessment (DAWBA;* Goodman et al., 2000)

This standardised diagnostic instrument is designed to generate ICD-10 and DSM 5 psychiatric diagnoses. The DAWBA provides a probability of the likelihood of a range of mental health disorders. The DAWBA incorporates the Strengths and Difficulties Questionnaire (SDQ), a brief (25-item) emotional and behavioural questionnaire and its impact supplement (Goodman, 1997). The DAWBA (Brøndbo et al., 2012) has been shown to have excellent reliability and validity.

Baseline and post-intervention measures

### Eating disorder psychopathology

*Eating Disorder Examination Questionnaire (EDE-Q; Fairburn & Beglin, 2008)*

CYP will be asked to complete the self-report version of the EDE-Q. The EDE-Q is a questionnaire which assesses core eating disorder-specific attitudes and behaviours (Bryant-Waugh et al., 1996). The EDE-Q derived from the widely used Eating Disorder Examination (Fairburn & Cooper, 1993), considered the gold standard for assessing eating disorder cognitions and behaviours. The EDE-Q consists of 28 items which assess the level of eating disorder psychopathology during the last 28 days, across four subscales: restrain (5 items), eating concern (5 items), weight concern (5 items) and shape concern (8 items). A global score is calculated from the average of the four subscales. Subscale items of the EDE-Q are rated on a 7-point scale, ranging from 0 (no restraint/concern) to 6 (restraint present everyday/extreme concern), thus higher scores indicate greater severity or frequency of eating disorder symptoms. The EDE-Q has good psychometric properties including good internal consistency, adequate test-retest reliability, good internal consistency, as well as high convergent and discriminant validity (Berg et al., 2012).

*Parent Eating Disorder Examination Questionnaire (PEDE-Q; Loeb, 2008)*

Parents will be asked to complete the parent-report version of the EDE-Q, known as the PEDE-Q. The PEDE-Q is a 29-item measure that assesses parental perceptions of the severity of their child's eating disorder symptoms. The PEDE-Q values the perspective of a parent or guardian, as often they have observed certain behaviors that a child will not report. There are four subscales within the PEDE-Q, including: Restraint, Eating Concern, Shape Concern, and Weight Concern. The PEDE-Q has good psychometric properties and provides incremental information that can aid in the assessment of adolescents with eating disorders (Drury et al., 2023).

*The Clinical Impairment Assessment (CIA; Bohn et al., 2008)*

The CIA is a 16-item, self-report questionnaire designed to measure psychosocial impairment due to eating disorder features in the past 28 days. Items are rated on a 4-point Likert-like scale, ranging from 0 (not at all) to 3 (a lot). The 16 items cover impairment in domains of life that are typically affected by eating disorder psychopathology: mood and self-perception, cognitive functioning, interpersonal functioning and work performance. A CIA global score is calculated as a severity index (ranging from 0 to 48), which higher scores indicating greater severity of clinical impairment. The CIA has demonstrated high levels of internal consistency, test-retest reliability, sensitivity to change, construct validity, and discriminant validity (Bohn et al., 2008; Reas et al., 2010). The CIA has been slightly modified to make it applicable to CYP (e.g., item 4 asks about school performance as opposed to work performance).

%Weight-for-height

CYP, and their parents/carers, will be asked to report on the CYP’s body weight and height so that their %weight-for-height (Weight x 100 reference weight for CYP of the same height) can be calculated.

Depression and anxiety symptomatology

*The Revised Child Anxiety and Depression Scale (RCADS; Chorpita et al., 2000)*

The self-report and parent-report versions of the RCADS will be used to measure the CYP’s anxiety and depression symptoms. It is a 47-item questionnaire for 8-18 year olds, which is sub-divided into six scales: separation anxiety disorder, social phobia, generalised anxiety disorder, panic disorder, obsessive compulsive disorder and low mood (major depression). Each item is scored on a 4-point Likert scale from 0 (never) to 3 (always). In addition to the subscales, the RCADS can be used to calculate a total anxiety score and total anxiety and depression score. The RCADS has good reliability on subscales and total scale, with internal consistency of adequate to excellent across the different subscales (Chorpita et al., 2005). The measure also has good test-retest reliability, and good convergent and concurrent validity (Chorpita et al., 2005; Donnelly et al., 2019).

If it is decided to no longer use the DAWBA following the pilot, families will be asked to complete the Strengths and Difficulties Questionnaire (SDQ) instead.

*The Strengths and Difficulties Questionnaire (SDQ; Goodman, 1997)*

The self-report and parent-report versions of the SDQ will be used to assess the CYP’s psychological wellbeing. The SDQ is brief (25-item) emotional and behavioural questionnaire for CYP aged 2-17 years of age. The 25 items comprise 5 scales of 5 items each, each rated on a 3-point Likert scale ranging from 0 (‘not at all’) to 2 (‘certainly true’). The scales include: emotional symptoms, conduct problems, hyperactivity/inattention, peer relationship problems, prosocial behaviour. The first four subscales can be summed to provide a total difficulties score. The SDQ also has an ‘impact supplement’ which assesses the functional impairment of the identified problems across four domains (school, home, friendships, leisure). Impact items are scored on a four-point scale from 0 (‘not at all’) to 2 (‘a great deal’). The reliability and validity of the SDQ has been established (Goodman, 2001; Muris et al., 2003).

Session-by-session measures throughout intervention

*Eating Disorder-15 for Youth (ED-15-Y; Accurso & Waller, 2021a; Accurso & Waller, 2021b)*

The ED-15-Y is a brief measure of eating disorder psychopathology over the past week for children and adolescents aged ≥8 years. The measure was adapted from the adult ED-15 (Tatham et al., 2015). The ED-15-Y includes 10 attitudinal items (measured on a 1-5 scale) and five behavioural items (frequency of dieting, binge eating, vomiting, laxative use, driven exercise), which are also rated on a five-point scale. The ED-15-Y has demonstrated excellent reliability, validity and sensitivity to change over time in CYP with eating disorders (Accurso & Waller, 2021).

*Goal Based Outcomes (GBOs; Law, 2006)*

The GBO scale is an idiographic outcome measure that is used to track progress during intervention. In their first guidance session, CYP, and/or their parents/carers, will be asked to identify up to 3 intervention goals relating to the CYP’s eating disorder difficulties. They will be asked to rate on a scale from 0-10 where they are in terms of achieving this goal; with 0 reflecting no progress towards the goal and 10 meaning that the goal has been fully reached. Families will then rate each goal as part of the routine outcome measures for each session. Goal-based outcomes have been shown to improve treatment retention, clinical outcomes and client progress (Delgadillo et al., 2018; Tryon et al., 2018).

*Outcome Rating Scale (ORS)/Child Outcome Rating Scale (CORS; Duncan et al., 2006)*

The ORS/CORS is a four-item scale that assesses areas of life functioning that change in response to therapeutic intervention: symptom distress, interpersonal wellbeing, social life, and overall wellbeing. Each dimension is displayed as a visual analogue scale ranging from 0 to 10 (10 = better functioning). The ORS is designed for children aged 13-18 years and the CORS can be completed by children aged 6-12 years; the CORS has the same format but contains more child friendly language and smiley and frowning faces. The ORS/CORS has good concurrent validity with other outcome measures, and high internal consistency and test-retest reliability (Duncan et al., 2006).

*Strengths and Difficulties Questionnaire Session by Session (SDS SxS; Hall et al., 2015)*

The SDQ SxS measure is a modified form of the SDQ Impact Supplement that is intended for intervention progress monitoring. Self-rated items assess young people’s improvement, impact of problems on everyday life in the present and anticipated improvement in the future.

CYP will be asked to provide their weight every week for risk and medical monitoring.

Post-treatment only measures

*The Strengths and Difficulties Questionnaire (SDQ; Goodman, 1997)*

The self-report and parent-report versions of the SDQ will be used to assess the CYP’s psychological wellbeing. The SDQ is brief (25-item) emotional and behavioural questionnaire for CYP aged 2-17 years of age. The 25 items comprise 5 scales of 5 items each, each rated on a 3-point Likert scale ranging from 0 (‘not at all’) to 2 (‘certainly true’). The scales include: emotional symptoms, conduct problems, hyperactivity/inattention, peer relationship problems, prosocial behaviour. The first four subscales can be summed to provide a total difficulties score. The SDQ also has an ‘impact supplement’ which assesses the functional impairment of the identified problems across four domains (school, home, friendships, leisure). Impact items are scored on a four-point scale from 0 (‘not at all’) to 2 (‘a great deal’). The reliability and validity of the SDQ has been established (Goodman, 2001; Muris et al., 2003).

*Acceptability questionnaire*

Families’ satisfaction with the intervention will be assessed using a seven-item questionnaire adapted from Creswell and colleagues (2010). CYP, and their parents/carers, will be asked to rate five areas of satisfaction on five-point Likert scales: change in the CYP’s disordered eating, satisfaction with the programme, the help received, overall satisfaction and whether they would recommend the approach to others. Families will also be asked about satisfaction with the amount of help received and the methods through which this was delivered. Families will also have the opportunity to provide qualitative feedback in response to open-ended questions assessing which elements of treatment participants found most and least helpful, and their suggestions for improvement (see [Appendix 3](#Appendix3)).

### Optional qualitative interview

All CYP, and their parents/carers, who start the treatment will be invited to attend separate optional qualitative interviews to explore their experience of receiving the intervention and acceptability of the treatment approach. Families who dropout of the study, and explicitly state that they do not wish to be contacted, will not be invited to take part.

Interviews will cover what families found helpful/unhelpful about the intervention, views on the mode, content and structure of the treatment and suggestions for improvement. An indicative topic guide can be found in [Appendix 4](#Appendix4). Qualitative interviews will be conducted over videocall or via the telephone by a member of the research team who did not deliver the treatment (e.g., an Honorary Research Assistant).

*Treatment completion/drop-out rates*

Information regarding rates of recruitment, retention, completion of measures and proportion of sessions attended by the participants will be recorded to examine feasibility.

## Data analysis

### Quantitative analysis

Statistical analyses will be performed using IBM SPSS Statistics 27. Descriptive analyses will be used to characterise the sample, to examine the participant flow through the study and attrition rates at each stage, and to assess satisfaction with the intervention.

Baseline and post-intervention measures

Symptom change will be measured by comparing scores at baseline and end of treatment. In order to ensure that the outcomes are not affected by attrition, the analyses will be conducted using Intention to Treat methods. Paired t-tests will be used to compare differences from the baseline measures to end of treatment, and Cohen’s *d* will be used to determine effect sizes (small effect ≥ 0.2, medium effect ≥ 0.5 and large effect ≥ 0.8).

Outcomes will also be evaluated on an individual level using Jacobson and Truax (1991)’s indices of meaningful change: Reliable Change Index (RCI) and Clinically Significant Change (CSC). Achievement of RCI indicates that the observed change is unlikely to be due to measurement error, and achievement of CSC indicates that change is substantial, relative to the clinical and non-clinical ranges of scores.

Session-by-session measures

Weekly measures (ED-15-Y, GBOs, ORS/CORS and SDQ SxS) will be analysed visually.

Acceptability

The feasibility and acceptability of the guided self-help intervention will be evaluated by analysing the treatment satisfaction of participants using both questionnaire measures and qualitative interviews.

### Qualitative analysis

Acceptability

Qualitative feedback interviews will be anonymised and transcribed verbatim. Reflexive thematic analysis will be used to analyse the data (Braun & Clarke, 2006). Reflexive thematic analysis consists of six iterative stages including i) data familiarisation; ii) data coding; iii) initial theme development; iv) theme refinement; v) defining themes; and vi) report write-up. Reflexive thematic analysis recognises the role of the researcher (i.e., their assumptions, values experiences etc.) in shaping the data analysis (Braun & Clarke, 2006). The analysis will be led by the study researcher, who will meet regularly with other study team members to discuss codes and themes in relation to the research themes to allow for alternative interpretations of the data. Braun and Clarke's (2023) guidance on producing and reporting methodologically thematic analysis will be used to ensure quality analysis.

# Intervention

The intervention is a modular, guided self-help intervention, based on the principles of Cognitive Behavioural Therapy (CBT). This intervention (and therapist manual) has been adapted from existing guided self-help interventions for adults with eating disorders (e.g., Fairburn, 2013; Schmidt, Treasure & Alexander, 2015) and CYP with anxiety (Creswell & Willetts, 2019), to be suitable for CYP with eating disorders. These adaptations have been informed by focus groups with CYP with lived experience of an eating disorder (*n* = 11), their parents/carers (*n =* 12), and health care professionals with experience in treating eating disorders in paediatric settings (*n* = 10). These interviews and focus groups explored these key stakeholders’ views and preferences towards the content, structure and modes of delivering guided self-help interventions for CYP with eating disorders.

The intervention will consist of 8 weekly guidance sessions that will be conducted with CYP. These sessions will be 30 minutes in duration. These sessions will take place via videocall or over the telephone with the guide (study researcher). Parents/carers are encouraged to attend.

The core aims of the intervention are to provide psychoeducational material provided as part of the cognitive-behavioural treatment of eating disorders for CYP (Dalle Grave & Calugi, 2020), and to encourage CYP to change a variety of thoughts and behaviours relating to eating, body weight and body shape. Families will have access to an online website that contains all of the module content, and also interactive PDFs where they can submit their reflections. Families will be asked to work through one module from the programme each week and will also be asked to complete some activities between each session. The intervention will be piloted with families and iterative improvements will be made to the intervention following their feedback.

The intervention will be supported by the study researcher, Emily Davey, who has over 3 years’ experience of working with CYP and families with common mental health difficulties in research settings. Each support session will focus on reviewing the previous weeks’ material and activities, with the aim of facilitating adherence to the intervention. The guide’s role will be to enhance motivation, troubleshoot problems and refer the family to the programme to enhance knowledge and skills usage. The support sessions are not intended to deliver new content.

Treatment will be overseen and supervised by Professor Roz Shafran (Clinical Psychologist), Dr Rachel Bryant-Waugh (Clinical Psychologist), Professor Nadia Micali (Child and Adolescent Psychiatrist) and Dr Sophie Bennett (Clinical Psychologist), who all have extensive experience in treating CYP with eating disorders and/or other common mental health difficulties (i.e., anxiety and depression).

**Intervention fidelity**

The intervention will be supported by Emily Davey, a postgraduate researcher with >3 years’ experience of working with children and young people with mental health difficulties in research settings. Emily will receive weekly supervision from members of her supervisory team (Roz Shafran, Sophie Bennett, Nadia Micali and Rachel Bryant-Waugh) throughout the research and intervention process to ensure fidelity to the agreed protocol. Guidance sessions will be audio/video-recorded and shared with the study supervisors to be discussed weekly in clinical supervision. Procedural fidelity during the intervention phase will also be monitored through the completion of adherence to the manual checklists after each guidance session.

# Discontinuation/Withdrawal of participants from study

**Discontinuation**

As weekly symptom tracking measures are being used, the guide and their supervisors will be able to quickly detect significant deterioration. If no progress and/or deterioration is seen, and this is not attributable to other known factors (e.g., a life event), the study team will discuss possible discontinuation with the family and referral to other more appropriate sources of support. If risk is identified during the study, this will be dealt with appropriately (see section on [Risk Management](#_Risk_management_1)).

**Withdrawal**

Participants have the right to withdraw from the study at any time, without impact on their clinical care. Participants who withdraw part-way through the study will be asked (under no obligation) to complete post-treatment measures. They will be asked whether they continue to consent/assent to being contacted for the qualitative interview. Data will be analysed on an intention to treat and last measure carried forward basis. Therefore, withdrawal from the study will not result in exclusion of the data for that participant from analysis.

# Ethical and safety considerations

The West of Scotland Research Ethics Committee 5 has granted ethical approval for this study, and ethical and safety considerations will be carefully monitored by the study research team.

**Time burden**

CYP and parents/carers will be required to complete various questionnaires and interviews throughout the study. The participant information sheets will provide clear information of what assessments will be involved and why, so that CYP and parents/carers understand the purpose of the assessments. Participants will be informed that they are free to withdraw from the study at any time they wish, without needing to provide a reason for withdrawal.

The screening questionnaire is likely to take around 15 minutes to complete. The baseline and post-intervention questionnaire measures approximately 25 minutes to complete. The baseline questionnaires up to 30 minutes to complete. The feedback appointment after the baseline questionnaires around 30 minutes. The weekly measures around 10 minutes to complete. The post-intervention acceptability questionnaire around 10 minutes to complete. The post-intervention questionnaires up to 30 minutes to complete. The post-intervention feedback appointment around 30 minutes. The optional qualitative interview around 45 minutes to complete.

Families will attend 8 x 30-minute guidance sessions with a Guide, and will also spend time reading through the intervention materials and completing the worksheets between sessions.

The inconvenience of completing questionnaires, assessments, interviews and the intervention will be minimised by offering appointment dates/times to suit the participants where possible. For example, participants will have the opportunity to complete measures electronically or over the telephone. Guidance sessions will similarly be offered over the telephone or via video call, and at a time most convenient for the family.

CYP will be provided with a £25 Amazon voucher for the additional time required to complete the questionnaires 12 weeks after completing the baseline measures. CYP, and their parents/carers, will be provided with a £25 voucher each if they decide to take part in the optional qualitative interview. CYP will therefore receive a maximum of £50 in vouchers and parents/carers a maximum of £25 in vouchers. This payment rate is in line with NIHR involve rates and reflective of the time and effort required of participants for the study.

**Potential for participant distress**

The screening questions, pre- and post-intervention measures, and weekly session-by-session measures will involve discussing thoughts and feelings that CYP, and their parents/carers, may find upsetting. These questions are similar to those used in routine clinical practice. Questionnaire measures and interviews will be conducted by a study researcher with experience in working with CYP with mental health difficulties, and their families. The researcher will be supervised by a team of clinical psychologists and a child and adolescent psychiatrist (with considerable experience working with families affected by mental health difficulties). If participants become distress during the questionnaires or interviews, participants will be reminded that they can take a break and/or withdraw from the study at any time.

Guidance sessions may also involve discussing thoughts and feelings that CYP, and their parents/carers, may find upsetting. Any distress will be managed by the study researcher with the support of the study supervisors, if necessary. CYP and/or their parents/carers (depending on who is attendance) will be reminded that they can take breaks during the guidance sessions and/or withdraw from the study at any time. It is possible that CYP, and their parents/carers, may find the treatment techniques, that they are implementing at home, upsetting. The study researcher will ensure that the treatment techniques are delivered to families in a step-by-step, gradual way to reduce the potential upset. The study researcher will have the support of the study supervisors to manage any distress.

The optional qualitative interviews may involve discussing thoughts and feelings that CYP, and their parents/carers, may find upsetting. The questions asked in the qualitative interviews will be discussed with PPI representatives and piloted before use to ensure that they are acceptable to families. The interviews will be conducted by a member of the study team with experience of working with families affected by mental health problems, who will have support from the study supervisors. CYP, and their parents/carers, will be reminded that they can take breaks during the interviews and/or withdraw from the study at any time.

The research team is made up of a number of clinically trained researchers, who will be well-placed to manage any distress should it arise, and to signpost/refer to sources of support (such as local mental health services) if necessary.

**Novel intervention**

CYP and parents/carers taking part in this study will be receiving an intervention which has been adapted from existing evidence-based interventions for adults with eating disorders, and CYP with anxiety. This adapted intervention has not yet been evaluated with CYP with eating disorders, and their parents/carers. As such, we cannot be sure what the outcomes of this intervention will be. We do not expect this treatment to cause adverse effects for participants based on the existing research conducted with adults with eating disorders, and CYP with anxiety.

The study researcher has over 3 years' experience working with families affected by mental health difficulties in research settings. The study researcher's role is to enhance motivation, troubleshoot problems and refer the family to the programme to enhance knowledge and skills usage. The researcher will not be introducing new content.

The study researcher will receive ongoing weekly supervision by the study supervisors (who have extensive experience working therapeutically with CYP with eating disorders and other common mental health difficulties) throughout the study. CYP's symptoms will be regularly measured throughout the intervention and any potentially harmful effects of the treatment will be reported immediately to the study supervisors. Participants will be given clear information regarding the treatment in the information sheets before giving consent/assent and the voluntary nature of the study will be clearly emphasised.

**Video/Audio-recordings**

Video and/or audio-recordings will be made during the guidance sessions and optional qualitative interviews. This may be considered intrusive or distressing to CYP and/or parent/carers. Participant information sheets will clearly state that the guidance sessions and the optional qualitative interviews will be video and/or audio-recorded and the reasons why these will be recorded. CYP (aged 16+) and parents/carers will be asked to provide explicit informed consent for video and/or audio-recordings to take place prior to participation in the study.

## Risk management

If risk is identified in the screening questionnaire, the family will be contacted by a trained clinician by a research team member for further assessment. The participant will not enrol in the study and any significant risk issues will be communicated to the families’ GP and any other clinician involved in their care.

If risk is identified throughout the intervention period, the family will be contacted by a research team member for further assessment. If the risk is significant, the family will be withdrawn from the study and risk information communicated to the families’ GP and any other clinician involved in their care. Referrals or requests for referrals to other services may be made as appropriate.

A risk and safeguarding management protocol that will be followed by the research team throughout the study can be found in [Appendix 5](#Appendix5).

# Data recording and record keeping

Measures will be completed online via a REDCap form, and anonymous data inputted into a statistical programme. Computerised data will be kept on an anonymous password-protected database. The participants will be identified by a unique participant ID in any database. The name and any other identifying detail will not be included in any study data electronic file.

# Confidentiality and information governance

All study data will be appropriately secured to meet governance requirements for collecting personal data. Identifiable patient data will be managed in accordance with the Caldicott Principles and Data Protection Act 2018, which requires data to be anonymised as soon as it is practical to do so.

**Confidentiality of personal data**

After providing informed consent, participants will be allocated a unique, sequential study identifier which will be used to label all study data. The document linking these numbers to participant details will be stored separately to study data, on a restricted access, secure drive on the Data Safe Haven. All electronic information will be kept secure and confidential in encrypted, password protected files stored on the UCL SLMS Data Safe Haven. The University College London (UCL) SLMS Data Safe Haven is a technical environment for receiving, handling and storing sensitive data securely. Data Safe Haven has been certified to the ISO27001 information security standard and conforms to NHS Digital’s Information Governance Toolkit.

Any paper records will be stored in locked filing cabinets in locked offices at UCL Great Ormond Street Institute of Child Health. Paper data will then be scanned and stored on the UCL Data Safe Haven and destroyed as confidential waste. No paper copies of personally identifiable data will be stored outside of the UCL Great Ormond Street Institute of Child Health offices.

Qualitative interviews will be de-identified at the point of transcription, and presentation of analyses will use direct, de-identified quotes from these transcripts. Qualitative analyses will also use direct, de-identified quotes from participants’ responses to open-ended treatment acceptability questions.

The research team will ensure that the participants’ anonymity is maintained, except where information is disclosed by a participant that suggests a risk of harm to themselves or others. Contact details collected from CYP, and parents/carers, who do not take part in the study will be securely destroyed.

**Access to personal data during the study**

Access to personally identifiable data will only be granted to appropriate researchers and clinical staff within the study team. The study supervisors (Professor Roz Shafran, Dr Sophie Bennett, Professor Nadia Micali and Dr Rachel Bryant-Waugh) will have access to personally identifiable information for the purposes of supervision only. Video and/or audio-recordings of guidance sessions will be shared with the study supervisors for supervision purposes only, either in in-person supervision or via a secure OneDrive link which can only be opened by the intended recipient. Personally identifiable information will only be shared with individuals outside the research team if the study supervisors recognise that this information needs to be shared with relevant professionals (e.g., GP, eating disorder clinician) in the event of a risk and/or safeguarding issue being identified. In such cases, the participant will be informed (where possible) that this information will be shared with the relevant professional.

**Data storage following study completion**

Identifiable information will be securely kept for 6 months following completion of the study. This excludes consent/assent forms, which will be held securely at UCL Great Ormond Street Institute of Child Health for 3 years after the end of the study and then securely destroyed. The document which links the participants’ ID to their details will be permanently destroyed at the end of the study. Audio/video recordings of the diagnostic assessments and treatment sessions will be stored until the end of the study. Audio/video recordings of the qualitative interviews will be stored until recordings have been transcribed verbatim and transcriptions thoroughly checked, which will be at the end of the study.

# Dissemination of results and publication policy

All individuals who have made substantial intellectual, scientific and practical contributions to the study and the manuscript will, where possible, be credited as authors.

The results of the study will be reported and disseminated as follows:

- Written feedback to participants;
- Peer reviewed scientific journals;
- Internal report, plus possible articles on Institute web pages (publicly accessible);
- Presentations at national, and potentially international, conferences

# Financial information and insurance

The study researcher, Emily Davey, holds a Child Health Research PhD Studentship at UCL Great Ormond Street Institute of Child Health. The time spent by Emily on this study and any study-related costs are therefore funded by this studentship.

University College London will provide standard indemnity insurance for the management and design of this research.

# References

Accurso, E. C., & Waller, G. (2021a). A brief session-by-session measure of eating disorder psychopathology for children and adolescents: Development and psychometric properties of the Eating Disorder-15 for Youth (ED-15-Y). *The International Journal of Eating Disorders*, *54*(4), 569–577. https://doi.org/10.1002/eat.23449

Accurso, E. C., & Waller, G. (2021b). Concordance between youth and caregiver report of eating disorder psychopathology: Development and psychometric properties of the Eating Disorder-15 for Parents/Caregivers (ED-15-P). *The International Journal of Eating Disorders*, *54*(7), 1302–1306. https://doi.org/10.1002/eat.23557

Ágh, T., Kovács, G., Supina, D., Pawaskar, M., Herman, B. K., Vokó, Z., & Sheehan, D. V. (2016). A systematic review of the health-related quality of life and economic burdens of anorexia nervosa, bulimia nervosa, and binge eating disorder. *Eating and Weight Disorders - Studies on Anorexia, Bulimia and Obesity*, *21*(3), 353–364. https://doi.org/10.1007/s40519-016-0264-x

Bennett, S. D., Cuijpers, P., Ebert, D. D., McKenzie Smith, M., Coughtrey, A. E., Heyman, I., Manzotti, G., & Shafran, R. (2019). Practitioner Review: Unguided and guided self-help interventions for common mental health disorders in children and adolescents: a systematic review and meta-analysis. *Journal of Child Psychology and Psychiatry*, *60*(8), 828–847. https://doi.org/10.1111/jcpp.13010

Bohn, K., Doll, H. A., Cooper, Z., O’Connor, M., Palmer, R. L., & Fairburn, C. G. (2008). The measurement of impairment due to eating disorder psychopathology. *Behaviour Research and Therapy*, *46*(10), 1105–1110. https://doi.org/10.1016/j.brat.2008.06.012

Braun, V., & Clarke, V. (2006). Using thematic analysis in psychology. *Qualitative Research in Psychology*, *3*(2), 77–101. https://doi.org/10.1191/1478088706qp063oa

Braun, V., & Clarke, V. (2023). Toward good practice in thematic analysis: Avoiding common problems and be(com)ing a knowing researcher. *International Journal of Transgender Health*, *24*(1), 1–6. https://doi.org/10.1080/26895269.2022.2129597

Brøndbo, H., Mathiassen, B., Martinussen, M., Heiervang, E., Eriksen, M., & Kvernmo, S. (2012). Agreement on Web-based Diagnoses and Severity of Mental Health Problems in Norwegian Child and Adolescent Mental Health Services. *Clinical Practice and Epidemiology in Mental Health : CP & EMH*, *8*, 16–21. https://doi.org/10.2174/1745017901208010016

Bryant-Waugh, R. J., Cooper, P. J., Taylor, C. L., & Lask, B. D. (1996). The use of the eating disorder examination with children: A pilot study. *International Journal of Eating Disorders*, *19*(4), 391–397. https://doi.org/10.1002/(SICI)1098-108X(199605)19:4<391::AID-EAT6>3.0.CO;2-G

Chorpita, B. F., Moffitt, C. E., & Gray, J. (2005). Psychometric properties of the Revised Child Anxiety and Depression Scale in a clinical sample. *Behaviour Research and Therapy*, *43*(3), 309–322. https://doi.org/10.1016/j.brat.2004.02.004

Chorpita, B. F., Yim, L., Moffitt, C., Umemoto, L. A., & Francis, S. E. (2000). Assessment of symptoms of DSM-IV anxiety and depression in children: A revised child anxiety and depression scale. *Behaviour Research and Therapy*, *38*(8), 835–855. https://doi.org/10.1016/S0005-7967(99)00130-8

Creswell, C., Hentges, F., Parkinson, M., Sheffield, P., Willetts, L., & Cooper, P. (2010). Feasibility of guided cognitive behaviour therapy (CBT) self‐help for childhood anxiety disorders in primary care. *Mental Health in Family Medicine*, *7*(1), 49–57.

Creswell, C., & Willetts, L. (2019). *Helping Your Child with Fears and Worries 2nd Edition: A self-help guide for parents* (P. P. Cooper & P. Waite, Eds.; 2nd edition). Robinson.

da Luz, F. Q., Swinbourne, J., Sainsbury, A., Touyz, S., Palavras, M., Claudino, A., & Hay, P. (2017). HAPIFED: A Healthy APproach to weIght management and Food in Eating Disorders: a case series and manual development. *Journal of Eating Disorders*, *5*(1), 29. https://doi.org/10.1186/s40337-017-0162-2

Dalle Grave, R., & Calugi, S. (2020). Cognitive Behavior Therapy for adolescents with Eating Disorders. Guilford Press.

Delgadillo, J., McMillan, D., Lucock, M., Leach, C., Ali, S., & Gilbody, S. (2014). Early changes, attrition, and dose–response in low intensity psychological interventions. *British Journal of Clinical Psychology*, *53*(1), 114–130. https://doi.org/10.1111/bjc.12031

Donnelly, A., Fitzgerald, A., Shevlin, M., & Dooley, B. (2019). Investigating the psychometric properties of the revised child anxiety and depression scale (RCADS) in a non-clinical sample of Irish adolescents. *Journal of Mental Health (Abingdon, England)*, *28*(4), 345–356. <https://doi.org/10.1080/09638237.2018.1437604>

Drury, C. R., Hail, L., Rienecke, R. D., Accurso, E. C., Coleho, J. S., Lock, J., Le Grange, D., & Loeb, K. L. (2023). Psychometric properties of the Parent Eating Disorder Examination Questionnaire. *International Journal of Eating Disorders, 56*(9), 1730-1742. <https://doi.org/10.1002/eat.23999>

Dumont, E., Jansen, A., Kroes, D., de Haan, E., & Mulkens, S. (2019). A new cognitive behavior therapy for adolescents with avoidant/restrictive food intake disorder in a day treatment setting: A clinical case series. *International Journal of Eating Disorders*, *52*(4), 447–458. https://doi.org/10.1002/eat.23053

Duncan, B. L., Sparks, J. A., Miller, S. D., Bohanske, R. T., & Claud, D. A. (2006). Giving Youth a Voice: A Preliminary Study of the Reliability and Validity of a Brief Outcome Measure for Children, Adolescents, and Caretakers. *Journal of Brief Therapy, 5*(2).

Fairburn, C. G., Brownell, K. D., Leahy, R. L., Wilson, G. T., & Striegel-Moore, R. H. (2013). *Overcoming Binge Eating: The Proven Program to Learn Why You Binge and How You Can Stop* (2nd edition). Guilford Press.

Fairburn, C. G., & Cooper, Z. (1993). The Eating Disorder Examination (12th edition). In *Binge eating: Nature, assessment, and treatment* (pp. 317–360). Guilford Press.

Goodman, R. (1997). The Strengths and Difficulties Questionnaire: A Research Note. *Journal of Child Psychology and Psychiatry*, *38*(5), 581–586. https://doi.org/10.1111/j.1469-7610.1997.tb01545.x

Goodman, R. (2001). Psychometric properties of the strengths and difficulties questionnaire. *Journal of the American Academy of Child and Adolescent Psychiatry*, *40*(11), 1337–1345. https://doi.org/10.1097/00004583-200111000-00015

Goodman, R., Ford, T., Richards, H., Gatward, R., & Meltzer, H. (2000). The Development and Well-Being Assessment: Description and initial validation of an integrated assessment of child and adolescent psychopathology. *Journal of Child Psychology and Psychiatry, and Allied Disciplines*, *41*(5), 645–655.

Hall, C. L., Moldavsky, M., Taylor, J., Marriott, M., Goodman, R., Sayal, K., & Hollis, C. (2015). Innovations in Practice: Piloting electronic session-by-session monitoring in Child and Adolescent Mental Health Services: a preliminary study. *Child and Adolescent Mental Health*, *20*(3), 171–174. https://doi.org/10.1111/camh.12066

Jacobson, N. S., & Truax, P. (1992). Clinical significance: A statistical approach to defining meaningful change in psychotherapy research. *Journal of Consulting and Clinical Psychology*, *59*(1), 12–19. https://doi.org/10.1037//0022-006x.59.1.12

Katzman, D. K. (2021). The COVID-19 Pandemic and Eating Disorders: A Wake-Up Call for the Future of Eating Disorders Among Adolescents and Young Adults. *Journal of Adolescent Health*, *69*(4), 535–537. https://doi.org/10.1016/j.jadohealth.2021.07.014

Kliem, S., Schmidt, R., Vogel, M., Hiemisch, A., Kiess, W., & Hilbert, A. (2017). An 8-item short form of the Eating Disorder Examination-Questionnaire adapted for children (ChEDE-Q8). *International Journal of Eating Disorders*, *50*(6), 679–686. https://doi.org/10.1002/eat.22658

Law D. (2006). *Goal based outcomes (GBOs): some useful information.* London: CORC.

Lock, J., Couturier, J., Matheson, B. E., Datta, N., Citron, K., Sami, S., Welch, H., Webb, C., Doxtdator, K., & John-Carson, N. (2021). Feasibility of conducting a randomized controlled trial comparing family-based treatment via videoconferencing and online guided self-help family-based treatment for adolescent anorexia nervosa. *International Journal of Eating Disorders*. <https://doi.org/10.1002/eat.23611>

Loeb, K. L. (2008).  *Eating disorder examination: Parent version*. Mount Sinai Medical School.

Muris, P., Meesters, C., & van den Berg, F. (2003). The Strengths and Difficulties Questionnaire (SDQ). *European Child & Adolescent Psychiatry*, *12*(1), 1–8. https://doi.org/10.1007/s00787-003-0298-2

National Audit Office. (2023). Progress in improving mental health services in England. Retrieved from https://www.nao.org.uk/wp-content/uploads/2023/02/Progress-in-improving-mental-health-services-CS.pdf. [Last accessed 8 March 2023].

National Collaborating Centre for Mental Health. (2015). Access and waiting time standard for children and young people with an eating disorder. Retrieved from <https://www.england.nhs.uk/wp-content/uploads/2015/07/cyp-eating-disorders-access-waiting-time-standard-comm-guid.pdf>. [Last accessed 8 March 2023.

National Institute for Health and Care Excellence (2017). *Eating disorders: Recognition and Management* [NICE Guideline No. 69]. <https://www.nice.org.uk/guidance/ng69> [Last accessed 8 March 2023].

NHS England. (2023). Children and young people with an eating disorder waiting times. <https://www.england.nhs.uk/statistics/statistical-work-areas/cyped-waiting-times/> [Last accessed 8 March 2023].

Newlove-Delgado. (2022). *Mental Health of Children and Young People in England 2022—Wave 3 follow up to the 2017 survey*. https://digital.nhs.uk/data-and-information/publications/statistical/mental-health-of-children-and-young-people-in-england/2022-follow-up-to-the-2017-survey

Reas, D. L., Rø, O., Kapstad, H., & Lask, B. (2010). Psychometric properties of the clinical impairment assessment: Norms for young adult women. *The International Journal of Eating Disorders*, *43*(1), 72–76. https://doi.org/10.1002/eat.20653

Santomauro, D. F., Melen, S., Mitchison, D., Vos, T., Whiteford, H., & Ferrari, A. J. (2021). The hidden burden of eating disorders: An extension of estimates from the Global Burden of Disease Study 2019. *The Lancet Psychiatry*, *8*(4), 320–328. https://doi.org/10.1016/S2215-0366(21)00040-7

Schmidt, U., Lee, S., Beecham, J., Perkins, S., Treasure, J., Yi, I., Winn, S., Robinson, P., Murphy, R., Keville, S., Johnson-Sabine, E., Jenkins, M., Frost, S., Dodge, L., Berelowitz, M., & Eisler, I. (2007). A Randomized Controlled Trial of Family Therapy and Cognitive Behavior Therapy Guided Self-Care for Adolescents With Bulimia Nervosa and Related Disorders. *Am J Psychiatry*, *164*, 4.

Schmidt, U., Treasure, J., & Alexander, J. (2015). *Getting better bite by bite: A survival kit for sufferers of bulimia nervosa and binge eating disorders* (2nd edition)*.* Routledge

Skivington, K., Matthews, L., Simpson, S. A., Craig, P., Baird, J., Blazeby, J. M., Boyd, K. A., Craig, N., French, D. P., McIntosh, E., Petticrew, M., Rycroft-Malone, J., White, M., & Moore, L. (2021). A new framework for developing and evaluating complex interventions: Update of Medical Research Council guidance. *BMJ*, *374*, n2061. https://doi.org/10.1136/bmj.n2061

Spigel, R., Lin, J. A., Milliren, C. E., Freizinger, M., Vitagliano, J. A., Woods, E. R., Forman, S. F., & Richmond, T. K. (2021). Access to care and worsening eating disorder symptomatology in youth during the COVID-19 pandemic. *Journal of Eating Disorders*, *9*(1), 69. https://doi.org/10.1186/s40337-021-00421-9

Striegel Weissman, R., & Rosselli, F. (2017). Reducing the burden of suffering from eating disorders: Unmet treatment needs, cost of illness, and the quest for cost-effectiveness. *Behaviour Research and Therapy*, *88*, 49–64. https://doi.org/10.1016/j.brat.2016.09.006

Tatham, M., Turner, H., Mountford, V. A., Tritt, A., Dyas, R., & Waller, G. (2015). Development, psychometric properties and preliminary clinical validation of a brief, session-by-session measure of eating disorder cognitions and behaviors: The ED-15. *International Journal of Eating Disorders*, *48*(7), 1005–1015. https://doi.org/10.1002/eat.22430

Thirlwall, K., Cooper, P. J., Karalus, J., Voysey, M., Willetts, L., & Creswell, C. (2013). Treatment of child anxiety disorders via guided parent-delivered cognitive–behavioural therapy: Randomised controlled trial. *The British Journal of Psychiatry*, *203*(6), 436–444. https://doi.org/10.1192/bjp.bp.113.126698

Treasure, J. (2020). Eating disorders. *Medicine*, *48*(11), 727–731. https://doi.org/10.1016/j.mpmed.2020.08.001

Tryon, G. S., Birch, S. E., & Verkuilen, J. (2018). Meta-analyses of the relation of goal consensus and collaboration to psychotherapy outcome. *Psychotherapy*, *55*, 372–383. https://doi.org/10.1037/pst0000170

van Hoeken, D., & Hoek, H. W. (2020). Review of the burden of eating disorders: Mortality, disability, costs, quality of life, and family burden. *Current Opinion in Psychiatry*, *33*(6), 521–527. https://doi.org/10.1097/YCO.0000000000000641

Watkins, B., Frampton, I., Lask, B., & Bryant-Waugh, R. (2005). Reliability and validity of the child version of the eating disorder examination: A preliminary investigation. *International Journal of Eating Disorders*, *38*(2), 183–187. https://doi.org/10.1002/eat.20165

# Appendices

**Appendix 1a. Initial Screening Questionnaire for Parents/Carers**

About you

| Your name: |  |
| --- | --- |
| Your age: |  |
| Your gender: |  |
| Relationship to child: |  |

| Your email address: |  |
| --- | --- |
| Your phone number: |  |
| Your home address: |  |

| Are you a UK resident? | Yes/No |
| --- | --- |

Please enter the relevant code from the table below for you.

| Your ethnicity: |  |
| --- | --- |

| **Asian or Asian British** | **Code** | **White** | **Code** |
| --- | --- | --- | --- |
| Indian  Pakistani  Bangladeshi  Chinese  Any other Asian background | A  B  C  D  E | British  Irish  Any other White background | M  N  O |
| **Black or Black British** |  | **Other Ethnic group** |  |
| Caribbean  African  Any other Black, Black British, or Caribbean background | F  G  H | Any other Ethnic group | P |
| **Mixed or multiple ethic groups** |  | **Not stated** |  |
| White and Black Caribbean  White and Black African  White and Asian  Any other Mixed or multiple ethnic background | I  J  K  L | I do not wish to state ethnicity | Q |

| Do you have a significant intellectual impairment which would interfere with your ability to engage in treatment for your child? | Yes/No |
| --- | --- |

About your child

| Your child’s name: |  |
| --- | --- |
| Your child’s date of birth: |  |
| Your child’s gender: |  |

| Is your child a UK resident? |  |
| --- | --- |
| Does your child live at the same address as you? | Yes/No |
| If not, please state your child’s home address: |  |

| Your child’s GP name and contact details: |  |
| --- | --- |

| Is your GP the same as your child’s? | Yes/No |
| --- | --- |
| If no – please provide your GP name and contact details: |  |

Please enter the relevant code from the table below for your child.

| Your child’s ethnicity: |  |
| --- | --- |

| **Asian or Asian British** | **Code** | **White** | **Code** |
| --- | --- | --- | --- |
| Indian  Pakistani  Bangladeshi  Chinese  Any other Asian background | A  B  C  D  E | British  Irish  Any other White background | M  N  O |
| **Black or Black British** |  | **Other Ethnic group** |  |
| Caribbean  African  Any other Black, Black British, or Caribbean background | F  G  H | Any other Ethnic group | P |
| **Mixed or multiple ethic groups** |  | **Not stated** |  |
| White and Black Caribbean  White and Black African  White and Asian  Any other Mixed or multiple ethnic background | I  J  K  L | I do not wish to state ethnicity | Q |

About your child’s difficulties

The following questions will help us to understand if your child may have symptoms of an eating disorder which impair their functioning.

**Adapted version of Child Eating Disorder Examination Questionnaire (ChEDE-Q8; Kliem et al., 2017)**

| These questions are about the past two weeks only (14 days).  Please read each question carefully and circle the response for each question. | | Scoring | | | | | | |
| --- | --- | --- | --- | --- | --- | --- | --- | --- |
| On how many days of the past 14 days… | | No days | 1-2 days | 3-6 days | 7 days | 8-10 days | 12-13 days | Every day |
| Restraint over eating | ..has your child been trying to cut down on food to control their weight or shape? |  |  |  |  |  |  |  |
| Food avoidance | …has your child tried not to eat any foods they like to control their weight and shape? |  |  |  |  |  |  |  |
| Preoccupation with food | …has thinking about food or calories made it much harder for your child to concentrate on things they are interested  in; for example, reading, watching TV, or doing their homework? |  |  |  |  |  |  |  |
| Feelings of fatness | ..has your child felt fat? |  |  |  |  |  |  |  |
| Desire to lose weight | ..has your child had a very strong wish to lose weight? |  |  |  |  |  |  |  |
| Guilt about eating | Over the past 2 weeks/14 days: How often has your child felt guilty after eating because of the effect on their shape and weight?  (Do not count binges – times when your child has eaten a really big amount of food and felt out of control) |  |  |  |  |  |  |  |
| Dissatisfaction with weight | Over the past 2 weeks/14 days: How unhappy has your child felt about their weight? |  |  |  |  |  |  |  |
| Discomfort seeing body | Over the past 2 weeks/14 days How uncomfortable has your child felt seeing their body: for  example, in the mirror, in shop windows, when they undress  or when they have a bath or shower? |  |  |  |  |  |  |  |

**Adapted version of the Strengths and Difficulties Questionnaire (SDQ) impact supplement (Goodman, 1999)**

| Overall, do you think that your child has difficulties around eating, food, body shape and/or body weight? | No | Yes – minor difficulties | Yes – definite difficulties | Yes – severe difficulties |
| --- | --- | --- | --- | --- |

If you have answered “Yes”, please answer the following questions about these difficulties:

| How long have these difficulties been present? | Less than a month | 1-5 months | 6-12 months | Over a year |
| --- | --- | --- | --- | --- |

| Do the difficulties upset or distress your child? | Not at all | Only a little | Quite a lot | A great deal |
| --- | --- | --- | --- | --- |

| Do the difficulties interfere with your child’s everyday life in the following areas? | Not at all | Only a little | Quite a lot | A great deal |
| --- | --- | --- | --- | --- |
| Home life |  |  |  |  |
| Friendships |  |  |  |  |
| Classroom learning |  |  |  |  |
| Leisure activities |  |  |  |  |

| Do the difficulties put a burden on you or the family as a whole? | Not at all | Only a little | Quite a lot | A great deal |
| --- | --- | --- | --- | --- |

**Additional question regarding self-harm and suicidality**

| Is your child currently having thoughts about hurting themself or ending their life? | Yes/No |
| --- | --- |

About your child’s health

| 1. | Does your child have an eating disorder diagnosis? | Yes/No |
| --- | --- | --- |
|  | *If yes to 1 – please specify:* |  |
|  | *Which eating disorder your child has* | Anorexia Nervosa  Bulimia Nervosa  Binge Eating Disorder  Other Specified Feeding or Eating Disorder (OSFED) |
|  | *How old your child was when they were diagnosed with an eating disorder* |  |
|  | *Who diagnosed your child with an eating disorder (e.g., CAMHS, private treatment provider)* |  |
| 2. | Is your child currently receiving any psychological treatment for their eating disorder (e.g., family-based therapy, cognitive behavioural therapy, counselling etc.)? | Yes/No |
|  | *If yes to 3 – please specify:* |  |
|  | *What treatment your child is currently receiving (e.g., family-based therapy, cognitive behavioural therapy, counselling etc.)* |  |
|  | *The length and duration of the treatment (i.e., the number of sessions your child has received/will receive in total)* |  |
|  | *When you are due to finish this treatment (if known)* |  |
|  | *Where your child is receiving this treatment (e.g., CAMHS, private treatment provider)* |  |
| 3. | Is your child currently prescribed any medication for mental health difficulties (e.g., sertraline, fluoxetine)? | Yes/No |
|  | *If yes to 3 – please specify:* |  |
|  | *What medication your child has been prescribed* |  |
|  | *How long your child has been taking this medication at the current dosage* |  |
| 4. | Is your child currently on the waitlist for treatment for their eating disorder? | Yes/No |
|  | *If yes to 4 – please specify:* |  |
|  | *What treatment your child is waiting to receive (e.g., CBT, counselling, medication, not known)* |  |
|  | *Where your child is waiting for treatment (e.g., CAMHS, private treatment provider)* |  |
| 5. | Has your child previously received treatment for their eating disorder? | Yes/No |
|  | *If yes to 5 – please specify:* |  |
|  | *What treatment your child received (e.g., family-based therapy, cognitive behavioural therapy, counselling etc.)* |  |
|  | *The length and duration of the treatment (i.e., if a psychological therapy, the number of sessions your child received an over what time period or if medication, what medication and for how long were they taking this medication)* |  |
|  | *How old your child was when they received this treatment* |  |
|  | *Where your child received this treatment (e.g., CAMHS, private treatment provider)* |  |
| 6. | Has your child received a diagnosis for any other mental health condition? | Yes/No |
|  | *If yes to 6 – please specify*  What other mental health condition(s) your child has |  |

Other

| Is there any other information that you think is relevant that we haven’t asked? |  |
| --- | --- |

| How did you hear about this study? |  |
| --- | --- |

**Appendix 1b. Initial Screening Questionnaire for CYP over 16**

About you

| Your name: |  |
| --- | --- |
| Your date of birth: |  |
| Your gender: |  |

| Your email address: |  |
| --- | --- |
| Your phone number: |  |
| Your home address: |  |

| Your GP’s name and contact details: |  |
| --- | --- |

| Are you a UK resident? | Yes/No |
| --- | --- |

Please enter the relevant code from the table below for you.

| Your ethnicity: |  |
| --- | --- |

| **Asian or Asian British** | **Code** | **White** | **Code** |
| --- | --- | --- | --- |
| Indian  Pakistani  Bangladeshi  Chinese  Any other Asian background | A  B  C  D  E | British  Irish  Any other White background | M  N  O |
| **Black or Black British** |  | **Other Ethnic group** |  |
| Caribbean  African  Any other Black, Black British, or Caribbean background | F  G  H | Any other Ethnic group | P |
| **Mixed or multiple ethic groups** |  | **Not stated** |  |
| White and Black Caribbean  White and Black African  White and Asian  Any other Mixed or multiple ethnic background | I  J  K  L | I do not wish to state ethnicity | Q |

About your difficulties

The following questions will help us to understand if you may have symptoms of an eating disorder which impair your functioning.

**Child Eating Disorder Examination Questionnaire (ChEDE-Q8; Kliem et al., 2017)**

| These questions are about the past two weeks only (14 days).  Please read each question carefully and circle the response for each question. | | Scoring | | | | | | |
| --- | --- | --- | --- | --- | --- | --- | --- | --- |
| On how many days of the past 14 days… | | No days | 1-2 days | 3-6 days | 7 days | 8-10 days | 12-13 days | Every day |
| Restraint over eating | …have you been trying to cut down on food to control your weight or shape? |  |  |  |  |  |  |  |
| Food avoidance | …have you tried not to eat any foods you like to control your weight and shape? |  |  |  |  |  |  |  |
| Preoccupation with food | …has thinking about food or calories made it much harder to concentrate on things you are interested  in; for example, reading, watching tv, or doing your homework? |  |  |  |  |  |  |  |
| Feelings of fatness | …have you felt fat? |  |  |  |  |  |  |  |
| Desire to lose weight | …have you had a very strong wish to lose weight? |  |  |  |  |  |  |  |
| Guilt about eating | Over the past 2 weeks/14 days: How often have you felt guilty after eating because of the effect on your shape and weight?  (Do not count binges – times when you have eaten a really big amount of food and felt out of control) |  |  |  |  |  |  |  |
| Dissatisfaction with weight | Over the past 2 weeks/14 days: How unhappy have you felt about your weight? |  |  |  |  |  |  |  |
| Discomfort seeing body | Over the past 2 weeks/14 days How uncomfortable have you felt seeing your body: for  example, in the mirror, in shop windows, when you undress  or when you have a bath or shower? |  |  |  |  |  |  |  |

**Adapted version of the Strengths and Difficulties Questionnaire (SDQ) impact supplement (Goodman, 1999)**

| Overall, do you think that you have difficulties around eating, food, body shape and/or body weight? | No | Yes – minor difficulties | Yes – definite difficulties | Yes – severe difficulties |
| --- | --- | --- | --- | --- |

If you have answered “Yes”, please answer the following questions about these difficulties:

| How long have these difficulties been present? | Less than a month | 1-5 months | 6-12 months | Over a year |
| --- | --- | --- | --- | --- |

| Do the difficulties upset or distress you? | Not at all | Only a little | Quite a lot | A great deal |
| --- | --- | --- | --- | --- |

| Do the difficulties interfere with your everyday life in the following areas? | Not at all | Only a little | Quite a lot | A great deal |
| --- | --- | --- | --- | --- |
| Home life |  |  |  |  |
| Friendships |  |  |  |  |
| Classroom learning |  |  |  |  |
| Leisure activities |  |  |  |  |

| Do the difficulties make it harder for those around you (family, friends, teachers, etc.)? | Not at all | Only a little | Quite a lot | A great deal |
| --- | --- | --- | --- | --- |

**Additional question regarding self-harm and suicidality**

| Are you currently having thoughts about hurting yourself or ending your life? | Yes/No |
| --- | --- |

About your health

| 1. | Do you have an eating disorder diagnosis? | Yes/No |
| --- | --- | --- |
|  | *If yes to 1 – please specify:* |  |
|  | *Which eating disorder you have* | Avoidant/Restrictive Food Intake Disorder (ARFID)  Anorexia Nervosa  Bulimia Nervosa  Binge Eating Disorder  Other Specified Feeding or Eating Disorder (OSFED) |
|  | *How old were you when you were diagnosed with an eating disorder* |  |
|  | *Who diagnosed you with an eating disorder (e.g., CAMHS, private treatment provider)* |  |
| 2. | Are you currently receiving any psychological treatment for your eating disorder (e.g., family-based therapy, cognitive behavioural therapy, counselling etc.)? | Yes/No |
|  | *If yes to 3 – please specify:* |  |
|  | *What treatment you are currently receiving (e.g., family based therapy, cognitive behavioural therapy, counselling etc.)* |  |
|  | *The length and duration of the treatment (i.e., the number of sessions you have received/will receive in total)* |  |
|  | *When you are due to finish this treatment (if known)* |  |
|  | *Where you are receiving this treatment (e.g., CAMHS, private treatment provider)* |  |
| 3. | Are you currently prescribed any medication for mental health difficulties (e.g., sertraline, fluoxetine)? | Yes/No |
|  | *If yes to 3 – please specify:* |  |
|  | *What medication you have been prescribed* |  |
|  | *How long you have been taking this medication at the current dosage* |  |
| 4. | Are you currently on the waitlist for treatment for an eating disorder? | Yes/No |
|  | *If yes to 4 – please specify:* |  |
|  | *What treatment you are waiting to receive (e.g., CBT, counselling, medication, not known)* |  |
|  | *Where you are waiting for treatment (e.g., CAMHS, private treatment provider)* |  |
| 5. | Have you previously received treatment for an eating disorder? | Yes/No |
|  | *If yes to 5 – please specify:* |  |
|  | *What treatment you received (e.g., family based therapy, cognitive behavioural therapy, counselling etc.)* |  |
|  | *The length and duration of the treatment (i.e., if a psychological therapy, the number of sessions you received and over what time period or if medication, what medication and for how long were you taking this medication)* |  |
|  | *How old you were when you received this treatment* |  |
|  | *Where you received this treatment (e.g., CAMHS, private treatment provider)* |  |
| 6. | Have you received a diagnosis for any other mental health condition? | Yes/No |
|  | *If yes to 6 – please specify*  What other mental health condition(s) you have |  |

Other

| Is there any other information that you think is relevant that we haven’t asked? |  |
| --- | --- |

**Appendix 1c. Initial Screening Questionnaire for CYP under 16**

About you

| Your name: |  |
| --- | --- |
| Your parent/carer’s name: |  |
| Your parent/carer’s phone number: |  |
| Your parent/carer’s email address: |  |

About your difficulties

The following questions will help us to understand if you may have difficulties around eating which may get in the way of doing things.

**Child Eating Disorder Examination Questionnaire (ChEDE-Q8; Kliem et al., 2017)**

| These questions are about the past two weeks only (14 days).  Please read each question carefully and circle the response for each question. | | Scoring | | | | | | |
| --- | --- | --- | --- | --- | --- | --- | --- | --- |
| On how many days of the past 14 days… | | No days | 1-2 days | 3-6 days | 7 days | 8-10 days | 12-13 days | Every day |
| Restraint over eating | …have you been trying to cut down on food to control your weight or shape? |  |  |  |  |  |  |  |
| Food avoidance | …have you tried not to eat any foods you like to control your weight and shape? |  |  |  |  |  |  |  |
| Preoccupation with food | …has thinking about food or calories made it much harder to concentrate on things you are interested  in; for example, reading, watching tv, or doing your homework? |  |  |  |  |  |  |  |
| Feelings of fatness | …have you felt fat? |  |  |  |  |  |  |  |
| Desire to lose weight | …have you had a very strong wish to lose weight? |  |  |  |  |  |  |  |
| Guilt about eating | Over the past 2 weeks/14 days: How often have you felt guilty after eating because of the effect on your shape and weight?  (Do not count binges – times when you have eaten a really big amount of food and felt out of control) |  |  |  |  |  |  |  |
| Dissatisfaction with weight | Over the past 2 weeks/14 days: How unhappy have you felt about your weight? |  |  |  |  |  |  |  |
| Discomfort seeing body | Over the past 2 weeks/14 days How uncomfortable have you felt seeing your body: for  example, in the mirror, in shop windows, when you undress  or when you have a bath or shower? |  |  |  |  |  |  |  |

**Adapted version of the Strengths and Difficulties Questionnaire (SDQ) impact supplement (Goodman, 1999)**

| Overall, do you think that you have difficulties around eating, food, body shape and/or body weight? | No | Yes – minor difficulties | Yes – definite difficulties | Yes – severe difficulties |
| --- | --- | --- | --- | --- |

If you have answered “Yes”, please answer the following questions about these difficulties:

| How long have these difficulties been present? | Less than a month | 1-5 months | 6-12 months | Over a year |
| --- | --- | --- | --- | --- |

| Do the difficulties upset or distress you? | Not at all | Only a little | Quite a lot | A great deal |
| --- | --- | --- | --- | --- |

| Do the difficulties interfere with your everyday life in the following areas? | Not at all | Only a little | Quite a lot | A great deal |
| --- | --- | --- | --- | --- |
| Home life |  |  |  |  |
| Friendships |  |  |  |  |
| Classroom learning |  |  |  |  |
| Leisure activities |  |  |  |  |

| Do the difficulties make it harder for those around you (family, friends, teachers, etc.)? | Not at all | Only a little | Quite a lot | A great deal |
| --- | --- | --- | --- | --- |

**Additional question regarding self-harm and suicidality**

| Are you currently having thoughts about hurting yourself or ending your life? | Yes/No |
| --- | --- |

**Appendix 2. Additional demographic questionnaire for parents/carers at baseline**

About you

| Caregiver status: | Primary caregiver  Secondary caregiver  Shared caregiver  Other |
| --- | --- |
| Relationship status: | Single, never married or civil partnered  Living with partner  Married or civil partnered  Divorced  Separated  Widowed  Not applicable |
| Highest level of education: | School completion  Further education (e.g., college, vocational courses)  Higher education (undergraduate degree)  Postgraduate qualification |
| Employment status: | Unemployed  Employed full-time  Employed part-time  Retired  Student |
| *If employed, please state occupation:* |  |

About you and your health

| 1. | Do you have personal lived experience of an eating disorder? | Yes/No |
| --- | --- | --- |
|  | *If yes to 1 – Have you sought help for this?* | Yes/No |
| 2. | Do you have personal lived experience of another mental health condition? | Yes/No |
|  | *If yes to 2 – Have you sought help for this?* | Yes/No |

**Appendix 3. Treatment acceptability questionnaire (adapted from Creswell et al., 2010)**

|  |  | Strongly disagree | Disagree | Neither agree or disagree | Agree | Strongly agree |
| --- | --- | --- | --- | --- | --- | --- |
| 1. | Overall, I am satisfied with the treatment I received |  |  |  |  |  |
| 2. | I am satisfied with the programme I received |  |  |  |  |  |
| 3. | I am satisfied with the support I received from the guide |  |  |  |  |  |
| 4. | I am satisfied with the number of support sessions I received |  |  |  |  |  |
| 5. | I am satisfied with the length of the support sessions I received |  |  |  |  |  |
| 6. | I am satisfied with the outcomes of the treatment I received |  |  |  |  |  |
| 7. | I would recommend this treatment approach to other families |  |  |  |  |  |

| 8. What aspects of this treatment did you like the most? |
| --- |
|  |

| 9. What aspects of this treatment did you like the least? |
| --- |
|  |

| 10. How can we improve this treatment for families in the future? |
| --- |
|  |

| 11. Please provide any other feedback or comments |
| --- |
|  |

*Adapted from: Creswell, C., Hentges, F., Parkinson, M., Sheffield, P., Willetts, L., & Cooper, P. (2010). Feasibility of guided cognitive behaviour therapy (CBT) self‐help for childhood anxiety disorders in primary care. Mental Health in Family Medicine, 7(1), 49–57.*

**Appendix 4. Indicative Topic Guide – Qualitative post-intervention feedback interview**

*Please note that the questions below are examples of the types of questions that children, young people and parents/carers might be asked post-intervention (with the wording of the questions being modified accordingly). The interview will be semi-structured and by nature, flexible in response to individual needs.*

Aim: To explore CYP, and parents/carers, experiences of the guided self-help intervention, with a particular focus on acceptability

1. **Introduction**

Establish the purpose of the interview and the limits of confidentiality

1. **Contextual information**

Review relevant contextual information with CYP or parent/carer. For example:

- Age of child
- Diagnoses
- Number of support sessions received
- Who attended the support sessions

1. **Initial views of the treatment approach (e.g., when they first heard about the study, read the participant information sheet and/or spoke to the study researcher)**

- What were your initial views of this treatment?
- What were your initial thoughts on the possible benefits/challenges of this approach?
- What were your views on how the study information was presented?
- Was there anything that you found particularly helpful/unhelpful at this stage?
- Was there anything else you would have liked at this stage?

1. **Experiences of receiving the treatment**

- Did your views of the treatment approach change once you started treatment?
- What did you like/dislike about the treatment approach?
- Probe for benefits for the CYP/parent/family
- Probe for challenges for the CYP/parent/family
- How did you/your child find implementing the treatment techniques at home?
- How did you/your child respond to this approach?

1. **Relevance of treatment to family**

- Do you feel that the treatment has had any positive/negative impact on the difficulties it was aimed at helping with?
- Explore what the particular difficulties being targeted
- Probe what has improved for the CYP
- Probe what has not improved for the CYP
- Do you think the issues covered in the treatment were relevant to the family?
- Probe what issues were relevant and why
- Probe what issues were not relevant and why

1. **Experiences of the mode, content and structure of the treatment (programme and sessions)**

- How did you/your child find the treatment programme?
- Probe for views on content, style, length
- Probe for any suggestions of things they would have found helpful/preferred to receive
- How did you/your child find completing the module activities?
- Explore understanding of tasks
- Probe for views on content, style, length
- Probe for any suggestions of things they would have found helpful/preferred to receive
- How did you/your child find the support sessions?
- Probe for views on content, frequency, duration, rapport
- Probe for whether they felt sufficiently supported to implement the strategies from the programme
- Probe for any suggestions of things they would have found helpful/preferred to receive

1. **Broader acceptability of the approach**

- Would you recommend this approach to other families?
- Probe for reasons why/why not
- Were there any issues that were not covered in the treatment that you wish were covered?
- Probe what would have been helpful to cover
- Probe what impact they think covering these issues would have had
- What would you change about this approach?
- What would you have liked more/less of with this approach?
- Do you have any suggestions for improvement to the treatment?
- How have you found continuing to implement the techniques now the sessions have ended?

1. **Summarise the interview**

Probe whether there is anything else that the CYP or parent wishes to add or comment on. Thank the person for their time.

**Appendix 5. Risk and Safeguarding Management Protocol**

1. **Introduction**

The guidelines outlined here apply to the management of risk and safeguarding issues that arise in the context of work carried out with families for this research project. All risk and safeguarding issues will be managed in accordance with relevant national polices and guidelines (see section 3).

1. **Research Activities**

The study researchers will engage in activities where potential risk and/or safeguarding issues may arise. These include:

- Unstructured telephone contacts with participants or potential participants (e.g., explaining study procedures, arranging appointments)
- Structured telephone and/or videocall contacts to conduct questionnaires and/or qualitative interviews with participants
- Written information received from participants or potential participants (e.g., emails, completed questionnaires)
- Guidance sessions conducted with participants

## Definition of risk and safeguarding

Risk refers to (i) risk to self; (ii) risk to others; (iii) risk from others; (iv) neglect to self, and (v); neglect from others. Safeguarding refers to the welfare of an individual and/or ensuring individuals are protected from harm. The study researchers have a responsibility to respond to risk and/or safeguarding information. Risk and/or safeguarding information may be in relation to the study participants (e.g., child, young person, parent) and/or someone else.

## Procedures for managing risk and/or safeguarding concerns

The procedures for managing risk and/or safeguarding concerns are outlined below.

## *4.1. Unstructured contact with participants and/or potential participants (e.g., to explain study procedures, to arrange appointments)*

Whenever the study researchers have an unstructured contact with a participant or potential participant, there is a chance that this person may disclose risk and/or safeguarding information.

The study researchers will have access to support from the study supervisors (Professor Roz Shafran and Dr Sophie Bennett, who are both Clinical Psychologists) when making unstructured contact with families, in the event risk and/or safeguarding information arises, and action needs to be taken. The study researchers will only make unstructured contacts with participants or potential participants between the hours of 8am and 8pm Monday to Friday so that one of the study supervisors will be available to contact via email and/or their mobile phones if needed.

In the event that a risk and/or safeguarding concern arises during unstructured contacts with participants or potential participants, the study researchers will:

1. Thank the individual for sharing the information and find an early opportunity to explain that this information may need to be shared with the study supervisors and, if necessary, other relevant professionals (e.g., their GP, their eating disorder clinician). Note – an eating disorder clinician will only be contacted if the family remain on the eating disorder service’s caseload during their time within the study (i.e., when on waitlist for assessment/treatment), and if the study supervisors identify that an eating disorder clinician needs to be informed. The eating disorder clinician can then record this information on their electronic patient record system
2. Explain they would like to ask a few more questions about this information. The researcher will refer to the risk/safeguarding prompt sheet (see [Appendix 5a](#Appendix5a)) to assist in gathering an appropriate level of information to allow the study supervisors to make a decision about whether this information needs to be passed onto a relevant professional (e.g., their GP, their eating disorder clinician)
3. Ensure the research team have full contact details for the participant or potential participant and arrange a time for a member of the research team to contact them (by telephone) later that day to discuss the information further
4. Contact the study supervisors (RS or SB) by email and/or telephone to seek advice on how to proceed and any actions required
5. Follow any actions agreed with the study supervisors (RS or SB), including (when appropriate) contacting the participant or potential participant at the agreed time to gather further information and/or share planned actions
6. Document the risk/safeguarding information on the ‘Risk Management Log’ within 24 hours and save this on a secure file on the UCL Data Safe Haven. This can then be accessed by the study supervisors, RS and SB

*4.2. Structured contact with a participant to conduct questionnaires, and/or qualitative interviews*

The study researchers will not routinely ask risk questions during structured contacts with a participant (e.g., questionnaires, qualitative interviews). However, there is a chance that the study researchers will become aware of risk/safeguarding information.

The study researchers will have access to support from the study supervisors (Professor Roz Shafran and Dr Sophie Bennett, who are both Clinical Psychologists) when making structured contact with families, in the event risk and/or safeguarding information arises, and action needs to be taken. The study researchers will confirm a provisional time with families to conduct structured contacts (e.g., questionnaires, qualitative interviews). Before confirming this appointment time with families, the study researcher will contact the study supervisors (RS or SB) to confirm that at least one of the study supervisors will be available during and after the appointment to respond to any risk/safeguarding information as appropriate. Structured contacts may be scheduled to take place outside of usual working hours (e.g., 9am to 5pm Monday to Friday) as long as a study supervisor (RS or SB) has confirmed that they are available.

Prior to any structured contact with a participant, the study researchers will explain confidentiality and the limits of confidentiality. There should be a common understanding that if the participant shares information that indicates that they or someone else may be unsure or at risk of harm, the study researcher will share this information with the study supervisors (and if necessary, other relevant professionals e.g., their GP, their eating disorder clinician).

In the event that a risk and/or safeguarding concerns arises during structured contacts with participants or potential participants, the study researchers will:

1. Thank the individual for sharing the information and find an early opportunity to explain that this information may need to be shared with the study supervisors and, if necessary, other relevant professionals (e.g., their GP, their eating disorder clinician). Note – an eating disorder clinician will only be contacted if the family remain on the eating disorder service’s caseload during their time within the study (i.e., when on waitlist for assessment/treatment), and if the study supervisors identify that an eating disorder clinician needs to be informed. The eating disorder clinician can then record this information on their electronic patient record system
2. Explain they would like to ask a few more questions about this information. The researcher will refer to the risk/safeguarding prompt sheet (see [Appendix 5a](#Appendix5a)) to assist in gathering an appropriate level of information to allow the study supervisors to make a decision about whether this information needs to be passed onto a relevant professional (e.g., their GP, their eating disorder clinician)
3. Ensure the research team have full contact details for the participant or potential participant and arrange a time for a member of the research team to contact them (by telephone) later that day to discuss the information further
4. Contact the study supervisors (RS or SB) by email and/or telephone to seek advice on how to proceed and any actions required
5. Follow any actions agreed with the study supervisors (RS or SB), including (when appropriate) contacting the participant or potential participant at the agreed time to gather further information and/or share planned actions
6. Document the risk/safeguarding information on the ‘Risk Management Log’ within 24 hours and save this on a secure file on the UCL Data Safe Haven. This can then be accessed by the study supervisors, RS and SB

## *4.3. Written information received from participants or potential participants (e.g., email, completed questionnaires)*

It is possible that the study researchers may receive written information (e.g., email, completed questionnaires) from a participant or potential participant which raises risk/safeguarding concerns.

In the event that a risk/safeguarding concern is raised during written communication with a participant/potential participant, the study researchers will:

1. Contact the study supervisors (RS or SB) via email and/or telephone to see advice on how best to proceed and the actions required as soon as possible
2. Follow any agreed actions, including (where appropriate) contacting the participant/potential participant via telephone to seek further information
3. Document the risk/safeguarding information on the ‘Risk Management Log’ within 24 hours and save this on a secure file on the UCL Data Safe Haven. This can then be accessed by the study supervisors, RS and SB

## *4.4. Guidance sessions with participants*

## The guide will not routinely ask risk questions during guidance sessions with a participant. However, there is a chance that the study researchers will become aware of risk/safeguarding information.

The study researchers will have access to support from the study supervisors (Professor Roz Shafran and Dr Sophie Bennett, who are both Clinical Psychologists) when making structured contact with families, in the event risk and/or safeguarding information arises, and action needs to be taken. The study researchers will confirm a provisional date/time with families to have the guidance sessions. Before confirming this appointment time with families, the study researcher will contact the study supervisors (RS or SB) to confirm that at least one of the study supervisors will be available during and after the appointment to respond to any risk/safeguarding information as appropriate. Guidance sessions may be scheduled to take place outside of usual working hours (e.g., 9am to 5pm Monday to Friday) as long as a study supervisor (RS or SB) has confirmed that they are available.

Prior to any guidance sessions with participant, the guide will explain confidentiality and the limits of confidentiality. There should be a common understanding that if the participant shares information that indicates that they or someone else may be unsure or at risk of harm, the study researcher will share this information with the study supervisors (and if necessary, other relevant professionals e.g., their GP, their eating disorder clinician).

The study researcher will receive weekly supervision with at least one of the study supervisors (RS, SB, Dr Rachel Bryant-Waugh or Professor Nadia Micali) to discuss treatment progress and any risk/safeguarding concerns.

In the event that a risk and/or safeguarding concerns arises during guidance sessions with participants or potential participants, the guide will:

1. Thank the individual for sharing the information and find an early opportunity to explain that this information may need to be shared with the study supervisors and, if necessary, other relevant professionals (e.g., their GP, their eating disorder clinician). Note – an eating disorder clinician will only be contacted if the family remain on the eating disorder service’s caseload during their time within the study (i.e., when on waitlist for assessment/treatment), and if the study supervisors identify that an eating disorder clinician needs to be informed. The eating disorder clinician can then record this information on their electronic patient record system
2. Explain they would like to ask a few more questions about this information. The researcher will refer to the risk/safeguarding prompt sheet (see [Appendix 5a](#Appendix5a)) to assist in gathering an appropriate level of information to allow the study supervisors to make a decision about whether this information needs to be passed onto a relevant professional (e.g., their GP, their eating disorder clinician)
3. Ensure the research team have full contact details for the participant or potential participant and arrange a time for a member of the research team to contact them (by telephone) later that day to discuss the information further
4. Contact the study supervisors (RS or SB) by email and/or telephone to seek advice on how to proceed and any actions required
5. Follow any actions agreed with the study supervisors (RS or SB), including (when appropriate) contacting the participant or potential participant at the agreed time to gather further information and/or share planned actions
6. Document the risk/safeguarding information on the ‘Risk Management Log’ within 24 hours and save this on a secure file on the UCL Data Safe Haven. This can then be accessed by the study supervisors, RS and SB

## Procedures to be followed by study supervisors when risk issues arise

1. One of the supervisors (who are Clinical Psychologists) will assess the level of clinical risk and make a decision about whether the information gathered is sufficient to make a decision about whether this information needs to be shared or not. In the event that information is not sufficient to make a decision about appropriate action, the study researcher or a supervisor will contact the parent/carer by telephone for further information
2. Where the supervisor assesses the level of risk indicates that this information needs to be shared, the participant will be contacted by telephone (by the study researcher or study supervisor) and advised that this information will be shared with the relevant professional (e.g., their GP, eating disorder clinician). Note – an eating disorder clinician will only be contacted if the family remain on the eating disorder service’s caseload during their time within the study (i.e., when on waitlist for assessment/treatment), and if the study supervisors identify that an eating disorder clinician needs to be informed. The eating disorder clinician can then record this information on their electronic patient record system. If it is not possible to contact the family, the information may need to be shared without their consent
3. If a relevant professional needs to be contacted (e.g., their GP, their eating disorder clinician), it is the responsibility of the study researcher to contact the relevant professionals (with the support of the study supervisors). It will be made clear within these communications that the research team do not hold clinical responsibility for the family so are passing on relevant information to those that do
4. All information surrounding risk and actions taken will be recorded in the ‘Risk Management Log’ within 24 hours and signed off by a study supervisor

## Procedures to be followed by study supervisors when safeguarding issues arise:

1. One of the supervisors (who are Clinical Psychologists) will assess whether the information surrounding the potential safeguarding issues gathered is sufficient to make a decision about whether this information needs to be shared or not. In the event that information is not sufficient to make a decision about appropriate action, the study researcher or supervisor will contact the participant by telephone for further information
2. Where the supervisor assesses the level of risk indicates that this information needs to be shared, the participant will be contacted by telephone (by the study researcher or study supervisor) and advised that this information will be shared with the relevant professional (e.g., their GP, their eating disorder clinician, local safeguarding team). Note – an eating disorder clinician will only be contacted if the family remain on the eating disorder service’s caseload during their time within the study (i.e., when on waitlist for assessment/treatment), and if the study supervisors identify that an eating disorder clinician needs to be informed. The eating disorder clinician can then record this information on their electronic patient record system. If it is not possible to contact the family, the information may need to be shared without their consent
3. Where safeguarding issues arise with families, advice should be sought (by the study researcher or study supervisor) from either the safeguarding lead at the eating disorder service in question and/or the family’s local area safeguarding lead as necessary
4. If it is deemed appropriate to make a safeguarding referral to a local safeguarding team, it is the responsibility of the study researcher (with support of the study supervisor and/or eating disorder clinician) to make this referral
5. All information surrounding safeguarding issues and actions taken will be recorded in the ‘Risk Management Log’ within 24 hours and signed off by a study supervisor

## National policies, guidelines and useful contacts

As we will be recruiting families across the UK, we will contact safeguarding services local to the individual family. Useful websites on safeguarding more broadly, include the NHS England website (<https://www.england.nhs.uk/safeguarding/>), the NCPCC website ([https://www.nspcc.org.uk/what-](https://www.nspcc.org.uk/what-you-can-do/report-abuse/) [you-can-do/report-abuse/](https://www.nspcc.org.uk/what-you-can-do/report-abuse/) and telephone number 0808 800 5000) and Child Line (0800 1111).

*Guides on information sharing*

https://webarchive.nationalarchives.gov.uk/20130321041141/https://[www.education.gov.uk/public](http://www.education.gov.uk/public) ations/eOrderingDownload/00807-2008BKT-EN-March09.pdf

*Guides on the management of self-harm and suicidal ideation*

http://webarchive.nationalarchives.gov.uk/20130107105354/<http://www.dh.gov.uk/prod_consum_> dh/groups/dh_digitalassets/@dh/@en/@ps/documents/digitalasset/dh_133492.pdf

*Guides on recognising and reporting suspected abuse and neglect*

<https://www.gov.uk/government/uploads/system/uploads/attachment_data/file/419604/What_to_> [do_if_you_re_worried_a_child_is_being_abused.pdf](https://www.gov.uk/government/uploads/system/uploads/attachment_data/file/419604/What_to_do_if_you_re_worried_a_child_is_being_abused.pdf)

## Appendix 5a. Risk and Safeguarding Prompt Sheet

If a potential risk or safeguarding issue is disclosed, consider gathering the following information. Please note, these questions act as a guide, the research team should ask any further questions deemed appropriate to determine whether a relevant professional (e.g., their GP, their eating disorder clinician) should be informed.

## Confidentiality –

Remind the individual that this information will likely be shared with the study supervisors and other relevant professionals (e.g., their GP, their eating disorder clinician) if deemed necessary.

## Risk to self –

Where an individual expresses current and/or past **thoughts and/or actions towards not wanting to be here, suicidal ideation and/or self-harm**, consider gathering the following information:

- Content: What are the thoughts the person is having? Are they having any specific thoughts?
- Duration/intensity: How often do the thoughts happen? How long do the thoughts last? (i.e., fleeting vs. enduring thoughts) How intense are the thoughts? (i.e., from a scale of 0 to 10)
- Triggers: Are there any triggers for the thoughts? Are there any times of the day when the thoughts are more/less likely to happen?
- Intentions: Does the individual have any intentions to act on these thoughts? (i.e., from a scale of 0 to 10)
- Actions: Has the individual taken any actions towards these thoughts? If so, what actions have they taken?
- Management: How does the person manage the thoughts?

## Risk to others –

Where an individual expresses current and/or past **thoughts or actions towards harming others**, consider gathering the following information:

- Content: What are the thoughts the person is having? Are they having any specific thoughts?
- Duration/intensity: How often do the thoughts happen? How long do the thoughts last? (i.e., fleeting vs. enduring thoughts) How intense are the thoughts? (i.e., from a scale of 0 to 10)
- Triggers: Are there any triggers for the thoughts? Are there any times of the day when the thoughts are more/less likely to happen?
- Intentions: Does the individual have any intentions to act on these thoughts? (i.e., from a scale of 0 to 10)
- Actions: Has the individual taken any actions towards these thoughts? If so, what actions have they taken?
- Management: How does the person manage the thoughts?

## Risk from others –

Where an individual indicates current and/or past risk from someone else, consider gathering the following information:

- What: What is the risk?
- Who: Who is posing the risk?
- Where: Where is this person at risk?
- When: When did/will this risk arise?
- Management: Is this currently being managed?

## Neglect to self/neglect from others –

Where an individual indicates current and/or past neglect to self/neglect from others, consider gathering the following information:

- What: What is the risk?
- Who: Who is posing the risk?
- Where: Where is this person at risk?
- When: When did/will this risk arise?
- Management: Is this currently being managed?

## Safeguarding concerns

Where an individual indicates current and/or past safeguarding concerns, consider gathering the following information:

- What: What is the risk?
- Who: Who is posing the risk?
- Where: Where is this person at risk?
- When: When did/will this risk arise?
- Management: Is this currently being managed?

## Management:

Where risk and/or safeguarding issues arise, consider gathering information on the current management of these issues. For example:

- Does anyone else know about the risk/safeguarding concern (e.g., family member, professional)? If yes, consider use of the 4 W’s (What? Who? Where? When?)
