# Supplementary material for: Guided self-help treatment for children and young people with threshold and subthreshold eating disorders: A pilot study protocol
Source: PLoS One. 2024 Apr 16;19(4):e0301606. doi: 10.1371/journal.pone.0301606 (PMC11020482; doi:10.1371/journal.pone.0301606)
Supplement: Attachment — Submitted filename: renamed_d1a0d.docx [file pone.0301606.s005.docx]

# COMMENTS – Manuscript PONE-D-23-42547

Title: “Guided self-help treatment for children and young people with threshold and subthreshold eating disorders: A pilot study protocol”

**Important note: This review pertains only to ‘statistical aspects’ of the study and so ‘clinical aspects’ [like medical importance, relevance of the study, ‘clinical significance and** **implication(s)’ of the whole study, etc.] are to be evaluated [should be assessed] separately/independently. Further please note that any ‘statistical review’ is generally done under the assumption that (such) study specific methodological [as well as execution] issues are perfectly taken care of by the investigator(s). This review is not an exception to that and so does not cover clinical aspects {however, seldom comments are made only if those issues are intimately / scientifically related & intermingle with ‘statistical aspects’ of the study}. Agreed that** **‘statistical methods’ are used as just tools here, however, they are vital part of methodology [and so should be given due importance]. I look at the manuscript in/with statistical view point, other reviewer(s) look(s) at it with different angle so that in totality the review is very comprehensive. However, there should be efforts from authors side to improve (may be by taking clues from reviewer’s comments). Therefore, please do not limit the revision only (with respect) to comments made here.**

**COMMENTS:** There are very few issues/observations about which I have different opinion [mainly they are suggestions for further larger study (If the intervention is shown to be effective in this pilot study, a fully powered randomised controlled trial will be warranted with the ultimate goal of increasing access to psychological treatment for children and young people threshold and subthreshold eating disorders)]. Such observations/concerns are given below:

This study being ‘pilot’ (feasibility study) in nature, sample size is not a big issue. However, as mentioned in line 264 [This pilot study aims to recruit up to 10 eligible children and young people] the sample size intended to be used for this study is questionably small {as unlikely to get fair idea of attrition rates at each stage as said in line 338 and also unlikely to yield/produce an estimate of effect size which can be used to design a fully powered randomised controlled trial” as said in lines 386-88}. Further, though many things are ignored (loosely looked at / evaluated)] in case of ‘pilot studies’, methodological issues need to be very rigorously followed {in contrast with / despite often quote: “Pilot (Proof of Concept) studies typically involve a small number of subjects, as well as more latitude [i.e., leeway, freedom, liberty] in statistical requirements”}.

I request authors to read following note pasted from one famous standard textbook on ‘Medical Research Methodology’ [though I am sure that these learned authors already know these things] as it is very essential to keep the limitations in mind while interpreting results {note that I am not asking you to change the design of this pilot study}.

For a pilot study it is alright to have ‘single-arm design’, or it is alright when that is the only possibility’, however, it is very essential to keep the limitations in mind while interpreting results. Further, note that a classical/ideal clinical trial/study needs/requires a concurrently {but similarly} handled/treated appropriately selected/chosen control/comparison parallel group/arm.

Mind you further that any “Inferential statistics (i.e., hypothesis testing + estimation of CI) is built on the population model [which means the underlying assumption is that there is/are population(s) and we are dealing with random sample(s) drawn from that/those population(s)]. Although in clinical trial (involving at least two groups) we do not really deal with random samples (generally a non-probabilistic convenience sampling), ‘allocation’ to treatment groups is ‘randomly’ done which enable us to evoke the population model and we can use inferential statistics safely. But when there is only one group (so that there is no question of random allocation), with ‘non-random’ selection, it may be questionable to use inferential statistics even if you have two measurement sets as ‘pre-post’ or many repeated measurements or use ‘internal grouping for comparison.”

While planning larger study you may consider using ‘wait listed’ control group. Though the measures/tools used (in this pilot phase and few may be used in larger study) are appropriate, most of them are likely to yield data that are in ‘ordinal’ level of measurement [and not in ratio level of measurement for sure {as the score two times higher does not indicate presence of that parameter/phenomenon as double (for example, a Visual Analogue Scales VAS score or say ‘depression’ score)}]. Then application of suitable non-parametric (or distribution free) test(s) is/are indicated/advisable [even if distribution may be ‘Gaussian’ (also called ‘normal’)]. Agreed that there is/are no non-parametric test(s)/technique(s) available to be used as alternative in all situation(s), but should be used whenever/wherever they are available.

Therefore, in short use suitable non-parametric test(s)/technique(s) while dealing with data that are in ‘ordinal’ level of measurement even if [despite that] the distribution may be ‘Gaussian’. Testing ‘normality’ in sample [by using any normality test(s)} is not required/desired while dealing with data that are in ‘ordinal’ level of measurement [as most of the normality tests are not valid for ‘ordinal’ data].

On the backdrop of what is stated in ‘Abstract- Methods’ that “A mixed-method open pilot trial design will be used” please note that any regression techniques are not basically/originally developed for any sort of [between or within group(s)] comparison(s). Moreover, a

brief note on intervention designed may be expected/desirable/appreciated, by most of the readers, I guess. Except these few points, this manuscript is ‘excellent’ and therefore only ‘Minor revision’ is recommended.
